# The role of residual (veterinary) antibiotics in chemical exposome analysis: Current progress and future perspectives

**DOI:** 10.1111/1541-4337.70105

**Published:** 2025-02-04

**Authors:** Md Zakir Hossain, Max L. Feuerstein, Benedikt Warth

**Affiliations:** ^1^ Faculty of Chemistry, Department of Food Chemistry and Toxicology University of Vienna Vienna Austria; ^2^ Exposome Austria, Research Infrastructure and National EIRENE Node Vienna Austria

**Keywords:** exposome, human biomonitoring (HBM), liquid chromatography–mass spectrometry (LC–MS), mixture effects, veterinary drugs

## Abstract

Humans are exposed to a complex mixture of environmental and food‐related chemicals throughout their lifetime. Exposome research intends to explore the nongenetic, that is, environmental causes of chronic disease and their interactions comprehensively. Residual antibiotics can enter the human body through therapeutics, foods of animal origin, aquatic products, or drinking water. In the last decade, significant levels of residual antibiotics in human urine have been described, demonstrating frequent exposure throughout populations. To which extent they contribute to human health risks is debated. Human biomonitoring (HBM) aims to determine and quantify concentrations of xenobiotics in human specimens and provides the toolbox to monitor exposure to diverse chemical exposures. Due to their public health implications, priority‐listed xenobiotics are routinely monitored in the European Union and other countries. However, antibiotics, an important class of (food‐derived) xenobiotics, are still not systematically investigated for a better and more holistic understanding in the context of exposomics. This review provides a comprehensive summary of HBM research related to antibiotics, existing liquid chromatography–mass spectrometry (LC–MS)‐based analytical methods, and potential health risks caused by unintended exposure. Incorporating antibiotics into the chemical exposome framework through routine HBM using multiclass analytical methods will provide a better understanding of the toxicological or pharmacological mixture effects and, ultimately, the chemical exposome.

## 1 INTRODUCTION

Residual antibiotics due to medical, nutritional, and environmental exposure in humans and animals may pose a risk to public health (Wang, Yang, et al., 2018). Antibiotics are not only used to treat infections in humans and animals but also for prophylaxis in livestock farming. Globally, antibiotic use in the livestock industry likely exceeds total human antibiotic consumption (Van Boeckel et al., 2017). Humans are exposed to antibiotics through therapeutics, residues in foods of animal origin, and aquatic sources such as drinking water, surface water, and sediment (Wang, Yang, et al., 2018). Overuse and misuse of antibiotics can cause antibiotic resistance to human pathogens, which is an immense public health concern globally (Wang et al., 2021). Besides antibiotic resistance, recurrent exposure to antibiotics from food and drinking water may have a cumulative effect on developing a wide range of metabolic, immunological, and psychological conditions by altering the human microbiome (Blaser, 2016; Lurie et al., 2015; Yang et al., 2017). For instance, human exposure to antibiotics can lead to direct health hazards mediated by the gut microbiota including obesity, diabetes, asthma, or inflammatory bowel disease (Bailey et al., 2014; Lapin et al., 2015; Mikkelsen et al., 2015; Murphy et al., 2014; Theochari et al., 2018; Wang et al., 2015).

Human exposure to antibiotics is often assessed by questionnaire surveys and prescription examinations, which lack information on residual antibiotics, particularly from food and aquatic sources (Wang et al., 2014). Considerable research has been conducted to assess external exposure to antibiotics based on surveying the occurrence data in food and the environment. Besides the conventional approaches to assess exposure, there is a growing trend to evaluate exposure from residual antibiotics using human biomonitoring (HBM) by liquid chromatography–mass spectrometry (LC–MS). HBM is considered a pertinent tool to assess the exposure to environmental and food toxicants, identify novel exposure chemicals, evaluate trends and changing exposures, establish distribution patterns for populations, identify highly exposed vulnerable groups, determine effects of technological innovation on human exposure, conduct epidemiological studies, and evaluate the effectiveness of regulatory action (Yusa et al., 2015).

In analogy to the genome, the concept of the “exposome” was first introduced in 2005 by Wild to encompass the life course of all environmental exposures from conception onward (Wild, 2005). In contrast to the genome, the exposome is highly dynamic and requires complex approaches for accurate characterization and decoding of the external exposure interactions with the internal biological process and corresponding effects on human health. With the technological advancement and application of LC–MS, the exposome concept has been shaped in parallel to the mostly targeted and single chemical class‐centered approaches typically applied in HBM studies (Braun et al., 2022; Lai et al., 2024). With new multianalyte targeted LC–MS approaches, a wide range of known environmental exposures and their metabolites can be determined at an individual level, whereas nontargeted, high‐resolution mass spectrometry (HRMS) can be employed in chemical screening approaches or used to identify novel exposure chemicals and their biotransformation products of human metabolism (Marín et al., 2018; Oesterle et al., 2021). Despite their wider coverage, HRMS‐based analytical methods currently used for chemical exposome analysis are rather limited in sensitivity (Flasch et al., 2023; Vitale et al., 2021). A coordinated and systemic exposome initiative is urgently needed by utilizing nontargeted HRMS to profile the nongenetic factors involved in disease and health outcomes (Vermeulen et al., 2020).

The human exposome was estimated to contain over 400,000 chemicals, including 200,000 food constituents; 100,000 commercial products, environmental chemicals, and metabolites; 100,000 microbial metabolites; and 10,000 drugs and drug metabolites (Jones, 2016). While exposure to various dietary compounds may have beneficial health effects on human health, food intake also represents a key source of harmful xenobiotics such as pesticides, illicit drugs, pharmaceutical compounds, industrial and personal care products, and mycotoxins (Braun et al., 2022; Marín et al., 2018; Štefanac et al., 2021). Very recently, the European Union (EU) published a report for monitoring veterinary medicinal products in live animals and animal products (EFSA, 2024). Of the total targeted samples (*n* = 342,580), 67% were analyzed for veterinary drugs and contaminants and 0.14% were noncompliant in the “other veterinary drugs” category according to currently used regulatory limits (EFSA, 2024).

Notably, environmental and food‐related xenobiotics have the potential to interact with drugs and influence drug metabolism. Pharmacometabolomics is a branch of metabolomics that investigates drug interactions (i.e., drug–drug and drug–food) that play a role in drug efficacy, safety, and drug–exposome interactions (Beger & Flynn, 2016). The study of drug–exposome interactions, or pharmacoexposomics, is a new research area that sheds light on the interplay of the effects of medical drugs with chronic exposure to cocktails of environmental and food‐related chemicals. Recent advances in MS‐based nontargeted HRMS metabolomics and exposomics have the potential to systematically investigate these interactions for a wide range of drugs and environmental and food‐related chemicals at the same time (Pristner & Warth, 2020). A comprehensive understanding through the medical application of pharmacoexposomics, alone or in combination with pharmacogenomics, is expected to further extend the role of exposome research in advancing personalized medicine and healthcare.

**FIGURE 1:**
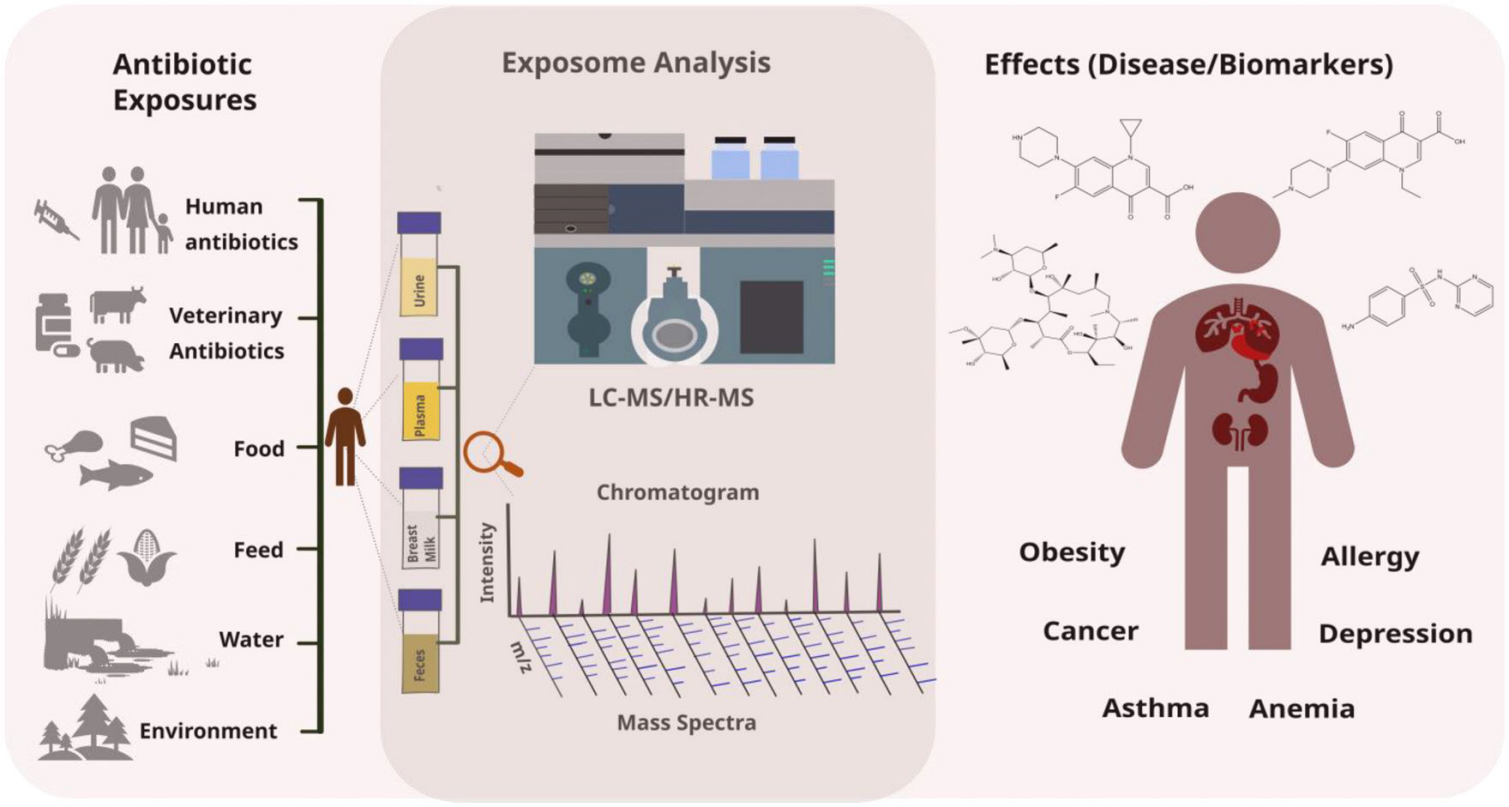
Exposome framework incorporating antibiotics for linking exposure assessed by omics‐scale analysis, potential health effects and related biomarkers.

Although veterinary antibiotics (VAs) represent a highly relevant class of xenobiotics, they are not systematically or routinely investigated within the human exposome domain. To date, a considerable number of targeted HBM studies were conducted to determine VA residues in human biofluids (Table 1), and a review on urinary biomonitoring of antibiotics and potential related adverse effects, including obesity, allergies, or mental disorders, has been published (Hu et al., 2022). However, a more comprehensive perspective on VA biomonitoring in various biofluids, including associated challenges and opportunities of its integration into a broader exposomics perspective, is lacking. Therefore, this review highlights the role of VAs in the context of the chemical exposome framework (Figure 1) and discusses analytical workflows suitable for integrating VAs in exposome analysis, including extraction protocols, sample cleanup procedures, and LC–MS‐based detection methods. Moreover, the results of existing HBM studies and the potential effects of VA exposure on human health are reviewed based on peer‐reviewed papers published since 2007.

**TABLE 1:** Overview of biomonitoring studies (*n* = 49) of residual antibiotics reported from 2007 to 2022.

Antibiotics/xenobiotics group	Number of antibiotics analyzed	Country	Biofluid	Sample preparation	Analytical method	Separation		Method performance				References
						Column	Mobile phase	Recovery (%)	RSD (%)	LOQ (ng/mL or ng/g)	LOD (ng/mL or ng/g)	
Aminoglycosides	5	Taiwan	Plasma	Protein precipitation	LC–Q‐ToF–MS, ESI	Vydac C18, 3 µm (Hesperia), ion pair chromatography	MP A: Water with 40 mM HFBA, MP B: MeOH/ACN	97–107	<6	n.p.	5000	Lu & Feng, 2007
Fluoroquinolones	15	Spain	Urine	Citrate buffer, no sample pretreatment	LC–UV/FLD	Zorbax Eclipse XDB‐C18 (150 × 4.6 mm, 5 µm)	MP: MeOH:ACN with 10 mM citrate buffer at pH 3.5 and 4.5	93–117	1.0–3.0	0.8–61	0.3–18	Canada‐Canada et al., 2007
β‐Lactams	5	Belgium	Plasma	SPE (Sep‐Pak cartridges, Waters), reconstituted phosphate buffer:ACN (95:5)	LC–UV	Symmetry C8 Column (250 × 4.6 mm, 5 µm)	MP A: ACN, MP B: Water (Phosphate buffer)	57–85	n.p.	500–1000	500	Denooz & Charlier, 2008
Sulfonamides, tetracyclines, penicillins, and amphenicols	11	Spain	Urine	MeOH: ACN or SPE (Bond Elut Plexa cartridges, Varian), Reconstitute: MeOH:ACN (1:1) with FA (0.1%)	LC–DAD and FLD	Gemini C18 Column (150 × 4.6 mm, 5 µm)	MP A: Water (0.1%FA), MP B: ACN (0.1% FA)	37–100	2.0–18.0	60–1060	10–320	Fernandez‐Torres et al., 2010
β‐Lactams	12	France	Plasma	ACN followed by chloroform	LC–UV	Xbridge C18 (30 × 4.6 mm, 2.5 µm, Waters)	Isocratic, MP group 1: can 8% (50 mM phosphate buffer), MP group 2: ACN 25% (100 mM phosphate buffer)	87–117	<8	2000–5000	1000–2000	McWhinney et al., 2010
Tetracyclines, oxytetracycline, and chlortetracycline	4	South Korea	Urine	EDTA, acidified with HCl, SPE (Oasis HLB), Reconstitute: ACN:water (1:9)	LC–Q‐ToF–MS, Z spray, and ESI	Acquity UPLC BEH C18 column (50 × 2.1 mm, 1.7 µm)	MP A: Water (0.1% FA), MP B: ACN (0.1% FA)	95–108	2.0–18	0.09–0.14	0.30–0.46	Jin et al., 2010
β‐Lactams	10	Australia	Plasma	Ultracentrifugation and stabilization	LC–PDA	Xbridge C18 (30 × 4.6 mm, 2.5 µm, Waters)	MP A: ACN (8%) with 50 mM phosphate buffer (92%), MP B: ACN (12%) with 50 mM phosphate (88%), MP A: ACN (25%) and 100 mM phosphate (75%), MP 2B: ACN (40%) and 100 mM phosphate buffer (60%)	n.p.	n.p.	100	n.p.	Briscoe et al., 2012
β‐Lactams	7	Belgium	Plasma	ACN (precipitate protein), ACN and dichloromethane (back‐ extraction)	LC–QqQ–MS	Acquity UPLC BEH C18 column (100 × 2.1 mm, 1.7 µm)	MP A: Water (0.1%FA), MP B: ACN (0.1% FA)	60–72	<15	500 (except piperacillin 1500)	n.p.	Carlier et al., 2012
Antibiotics, antifungals, antineoplastic, and antiviral	11	France	Plasma	Protein precipitation	LC–QqQ–MS, turbo ion spray, LC	Pentafluorophenyl (PFP) analytical column (50 × 2 mm, 2.6 µm, Phenomenex Kinetex)	MP A: Water (0.1% FA), MP B: MeOH (0.1%), MP C: Water and 10 mM ammonium formate with FA, MP D: ACN (0.1%FA)	79–106	3.0–9.0	0.03–1.8	n.p.	Jourdil et al., 2013
β‐Lactams	13	Belgium	Plasma	Mixed mode SPE using MCX sorbent	LC–QqQ–MS, ESI (orthogonal Y‐spray)	Acquity HSS T3 column (50 × 2.1 mm, 1.7 µm)	5% ACN and ACN (1 mM CH_3_COOH/CH_3_COONH_4_ buffer)	89–131	4.5–20	320–988	n.p.	Colin et al., 2013
Macrolides	5	South Korea	Urine	Dispersive liquid–liquid microextraction–solidification of floating organic droplets (DLLME‐SFO)	LC with Corona CAD (charged aerosol detection)	BDS C18 (250 × 4.6 mm, 5 µm)	MP A: Water (0.1% FA), MP B: ACN (0.1% FA)	94–118	0.4–13	100–250	10–40	Jia et al., 2013
β‐Lactams	7	Australia	Plasma	Protein precipitation with ACN and FA	LC–QqQ–MS, ESI	Kinetex C18 column (50 × 2.1 mm, 2.6 µm, Phenomenex)	MP A: Water (0.1% FA), MP B: MeOH (0.1% FA)	78–107	4–6.5	n.p.	100, except 250 (Flucloxacillin)	Sime et al., 2014
β‐Lactams, quinolones, tetracyclines, macrolides, sulfonamides, and chloramphenicol	14	China	Urine (children)	Enzymatic hydrolysis, Offline SPE (Oasis HLB), 96‐well	LC–Q‐ToF–MS, Z‐spray ESI	Xbridge C18 (30 × 2.1 mm, 10 µm), UPLC HSS T3 (100 × 2.1, 1.8 µm)	Water (0.1% FA), MeOH (0.1% FA)	80–121	3.0–18.0	n.p.	0.04–1.99	Wang et al., 2014
β‐Lactams	5	Belgium	Plasma	Protein precipitation	LC–QqQ–MS	Acquity UPLC BEH C18 column (100 × 2.1 mm, 1.7 µm)	MP A: Water (0.1% FA and 2 mM ammonium acetate), MP B: MeOH (0.1% FA and 2 mM ammonium acetate)	67–100	<11	520–1100	n.p.	Carlier et al., 2015
Antibiotics	3	United States	Plasma	Protein precipitation (0.1% TFA:ACN)	LC–QqQ–MS	ACE C18 column (100 × 3 mm)	FA:water:ACN (0.5:55:45)	90–100	<20			Winchester et al., 2015
Pesticides, veterinary drugs, and parabens	7	France	Urine	No sample preparation	LC–Q‐ToF–MS	Xselect CSH C18 (100 × 2.1 mm, 3.5 µm)	MP A: Water (0.1% FA and 10 mM NH₄HCO₂), MP B: ACN (0.1% FA and 10 mM NH₄HCO₂)	89–120	1.0–22.0	4.3–113.2	2.4–46	Cortejade et al., 2016
β‐Lactams including penicillin and cephalosporins	8	France	Plasma	Protein precipitation by ACN and FA (0.1%)	LC–UV	Hypersil Gold pentafluorophenyl column (100 × 2.1 mm, 1.9 µm)	MP A: Water (10 mM phosphoric acid), MP B: ACN	95–114	7.0–14.0	2000	n.p.	Legrand et al., 2016
Macrolides, β‐lactams, tetracyclines, fluoroquinolones, sulfonamides, and phenicols	21	China	Urine (children)	Enzymatic hydrolysis, SPE	LC–Q‐ToF–MS (Synapt G2), Z‐spray ESI	Xbridge C18 (30 × 2.1 mm, 10 µm), UPLC HSS T3 (100 × 2.1 mm, 1.8 µm)	MeOH:water (0.1%FA)	76–123	4.0–20.0	n.p.	0.01–0.05	Wang, Wang, Wang, Fang, et al., 2016
β‐lactams	3	Greece	Plasma and breast milk	Protein precipitation (ACN)	LC–Q–MS, ESI	ZIC‐HILIC analytical column (150 × 2.1 mm, 3.5 µm)	Isocratic, ACN (6% 12.5 mM ammonium acetate in water)	>96	<4	150–200	60–70	Kiriazopoulos et al., 2017
Sulfonamides, fluroquinolones, macrolides, tetracyclines, chloramphenicol, and β‐lactams	40	China	Serum	SPE (Oasis HLB)	LC–QqQ–MS, ESI	Xterra MS C18 column (100 × 2.1 mm, 3.5 µm)	MP A: Water (0.1% FA with 15.9 mmol/L ammonium acetate), MP B: MeOH:ACN (1:1)	41–132	25	n.p.	0.02–1.81	Liu et al., 2017
β‐Lactams	9	Netherlands	Plasma (blank)	Protein precipitation	LC–QqQ–MS, HESI Probe	Acquity UPLC BEH Amide Column C18 (100 × 2.1 mm, 1.7 µm, HILLIC)	ACN and 100 mM NH_4_HCO_2_ (8:2, pH 6.5)	100–155	2.0–46.0	50–750	n.p.	Abdulla et al., 2017
Tetracyclines, Sulfonamides, fluroquinolones, macrolides, and β‐lactams	13	China (Hong Kong)	Urine, food, and water (children)	SPE	LC–QqQ–MS, ESI	ZORBAX SB‐C18 (150 × 2.1 mm, 3.5 µm)	MP A: Water (0.1% FA), MP B: ACN	72–102	n.p.	0.01‐1	n.p.	Li et al., 2017
Contaminants including amphenicols, bisphenol analogs, and phthalate metabolites	16	China	Urine (children)	SPE (Oasis MAX cartridge)	LC–QqQ–MS, ESI	UPLC BEH C18 (50 × 2.1 mm, 1.7 µm) for bisphenols and amphenicols, UPLC CSH C18 (50 × 2.1 mm, 1.7 µm) for phthalates	MP A: Water (0.1%NH4OH), MP B: MeOH (0.1% NH4OH) & MP A: Water (0.1% CH_3_COOH), MP B: ACN (0.1% CH_3_COOH)	93.3–118	1.45–9.82	0.05–0.33	0.02–0.12	Yao et al., 2018
Antibiotics	5	Netherlands	Plasma (preterm infants)	SPE (Oasis HLB)	LC–QqQ–MS, ESI	Synergi fusion, RP18 column (50 × 4.6 mm, 2.5 µm)	MP A: Water (4% FA), MP B: MeOH (4% FA)	85–115	<11	120–380	n.p.	Chahbouni et al., 2015
Antibiotics	3	Serbia	Plasma	Protein precipitation (0.16% FA in ACN)	LC–QqQ–MS, SESI	ACQUITY Charge Surface Hybrid (CSH) C18 (50 × 2.1 mm, 1.7 µm, Waters)	MP A: Water (0.1% FA), MP B: ACN (0.1% FA)	85–115	15	0.05–0.18	0.02–0.09	Stajic et al., 2018
Antibiotics	5	Italy	Plasma	Dispersive LLME (liquid–liquid microextraction)	LC–PDA	Poroshell 120 SB C18 (50 × 2.1 mm, 2.6 µm)	MP A: Water (10 mM, pH 4 ammonium acetate), MP B: ACN:MeOH (80:20)	92–109	<10	n.p.	20–250	Ferrone et al., 2018
Antibiotics	18	China	Urine	SPE (Oasis HLB)	LC–Q‐ToF–MS (Synapt G2), Z‐spray ESI	UPLC HSS T3 (100 × 2.1 mm, 1.8 µm)	MP A: ACN, MP B: Water for phenicols; MP A: MeOH, MP 2B: Water (0.1% FA) for other antibiotics	78–117	8.0–16.0	0.13–4.37	0.04–1.31	Wang, Yang, et al., 2018
β‐Lactams, tetracyclines, macrolides sulfonamides, fluroquinolones, sulfonamides, and phenicols	20	China	Urine (children)	SPE (Oasis HLB)	LC–Q‐ToF–MS (Synapt G2), Z‐spray ESI	Xbridge C18 (30 × 2.1 mm, 10 µm), UPLC HSS T3 (100 × 2.1 mm, 1.8 µm)	Water (0.2% FA)	73–123	9.0–15.0	n.p.	0.04–1.87	Wang, Tang, et al., 2018
Fluoroquinolones	8	China	Urine and serum	SPE	LC–QqQ–MS	ZORBAX SB‐Aq C18 column (100 × 2.1 mm, 1.8 µm)	MP A: Water (0.1% FA), MP B: ACN	80–117	0.41–8	1.5–3	0.5–1	Wang et al., 2019
β‐Lactams, tetracycline, macrolides, trimethoprim, quinoxalines, sulfonamides, and quinolones	18	China	Urine	d‐SPE	LC–QqQ–MS	Gemini NX‐C18 column (50 × 2 mm, 3 µm)	MP A :Water (0.1%FA), MP B: MeOH (0.1% FA)	73–136 (macrolides: 52–78)	<15	0.38–7.38 (except 104.76: erythromycin)	0.11–1.86 (except 31.43: erythromycin)	Huang et al., 2019
Sulfonamides, fluoroquinolones, phenicols, macrolides, and tetracycline	15	China	Urine (pregnant women)	Enzymatic hydrolysis (β‐glucuronidase)	LC–QqQ–MS	Xbridge C18 (30 × 2.1 mm, 10 µm), UPLC HSS T3 (100 × 2.1 mm, 1.8 µm)	MeOH and water (0.1% FA): positive mode; ACN and water (0.1% FA): negative mode	85–115	<15	n.p.	0.1–1	Zeng et al., 2020
Macrolides, β‐lactams, tetracyclines, quinolones, sulfonamides, and aminoazoles	18	Tibet	Urine (school children)	d‐SPE	LC–QqQ–MS	Gemini NX‐C18 column (00B‐4453‐B0, 50 × 2 mm, 3 µm)	MP A: Water (0.1% FA), MP B: MeOH (0.1% FA)	73–136 (macrolides: 52%–78%)	<20	0.38–47.62 (except 104.76 :erythromycin)	0.11–14.29 (Except 31.43:erythromycin)	Huang et al., 2020
Fluoroquinolones, sulfonamides, β‐lactams, macrolides, tetracyclines, and phenicols	45	China	Urine (elderly population)	Oasis HLB SPE (60 mg, 3 mL, 30 µm),	LC–QqQ–MS	ZORBAX SB‐C18 (150 × 2.1 mm, 3.5 µm, Agilent)	MP A: Water (0.1% FA), MP B: ACN (0.1% FA)	73.5–112	<15	0.11–6.02	0.03–2.15	Zhu et al., 2020
Amoxicillin, clavulanic acid, benzylpenicillin, ciprofloxacin, trimethoprim, erythromycin, tetracycline, ceftriaxone, sulfamethoxazole, ampicillin, and metronidazole	12	Ghana	Urine	SPE	LC–QqQ–MS	Xterra MS C‐18 (100 × 2.1 mm, 2.5 µm)	MP A: Water (FA), MP B: ACN (FA)	71–125	<27	213–821	70–271	Bekoe et al., 2020
β‐Lactams including penicillin, cephalosporins, and carbapenem	8	France	Plasma and CSF	Protein precipitation (ACN)	LC–QqQ–MS,	Kinetex C18 (50 × 2.1 mm, 2.6 µm)	MP A: Water (0.1% FA), MP B: ACN (0.1% FA)	94–114	2–18.4	1000–2000	n.p.	Bellouard et al., 2020
Sulfonamides, macrolides, β‐lactams, tetracyclines, fluoroquinolones, phenicols, quinoxalines, and lincosamides	41	China	Urine (pregnant women)	SPE	LC–QqQ–MS	ZORBAX SB‐C18 (150 × 2.1 mm, 3.5 µm)	MP A: Water (0.1% FA), MP B: ACN (0.1% FA)	68–129	<13	0.01–1.48	0.05–4.81	Geng et al., 2020
Sulfonamides, macrolides, β‐ lactams, tetracycline, fluoroquinolones, phenicols, and quinoxalines	41	China	Urine (pregnant women)	HLB SPE	LC–QqQ–MS	ZORBAX SB‐C18 (150 × 2.1 mm, 3.5 µm)	MP A: Water (0.1% FA), MP B: ACN (0.1% FA)	76–113	1.0–11.1	0.05–4.81	0.02–1.48	Liu et al., 2020
Sulfonamides, fluoroquinolones, macrolides, β‐lactams, tetracyclines, and phenicols	45	China	Urine	Oasis HLB SPE (60 mg, 3 mL, 30 µm)	LC–QqQ–MS, ESI	XTerra MS C18 column (100 × 2.1 mm, 3.5 µm)	MP A: 95% water (0.1% FA), MP B: 5% MeOH/ACN	73.5–112	11	0.11–6.02	0.03–2.15	Zhang et al., 2020
Sulfonamides, tetracycline, fluoroquinolones, macrolides, and β‐lactams	19	China	Feces	SPE (SAX‐HLB)	LC–MS/MS	Acquity UPLC BEH C18 (100 × 2.1 mm, 1.7 µm)	MP A: ACN, MP B: Water (0.1% FA)	43–82	1.0–6.0	0.7–4	0.2–4.0	Wang et al., 2020
Tetracyclines, quinolones, macrolides, sulfonamides, phenicols, bisphenol A, and monobutyl phthalate	23	China	Urine (children)	SPE (Oasis HLB)	LC–Q‐ToF–MS (Synapt G2) Z‐spray ESI	CSH C18 (100 × 3 mm, 1.7 µm)	MeOH: Water (0.1%FA); ACN: Water (0.1% acetic acid)	72–113	n.p.	n.p.	0.01–2.8	Wang et al., 2021
Sulfonamides, fluoroquinolones, macrolides, β‐lactams, tetracyclines, and phenicols	45	China	Urine (children)	Oasis HLB SPE (60 mg, 3 mL, 30 µm),	LC–QqQ–MS, ESI	XTerra MS C18 column (100 × 2.1 mm, 3.5 µm, Waters)	MP A: 95% water (0.1% FA), MP B: 5% MeOH/ACN	74–112	n.p.	0.11–6.02	0.03–2.15	Zhang et al., 2021
Aminoglycoside	5	Japan	Plasma (healthy volunteer)	LLE	LC–Q‐ToF–MS, ESI	Multi‐mode ODS column (Imtakt Scherzo SM C18MF, 75 × 2 mm, 3 µm)	MP A: Water (50 mM Ammonium formate, pH 6), MP B: ACN:acetic acid (80:20)	93–120	1.5–7.4	1000–4000	500–2000	Minohara et al., 2021
Sulfonamides, quinolones, tetracyclines, macrolides, and phenicols	18	China	Urine	HLB cartridge (60 mg, 3 mL, 30 µm)	LC–QqQ–MS, ESI	Poroshell 120 EC‐C18 reversed‐phase column (100 × 3 mm, 2.7 µm, Agilent)	MP A: MeOH, MP B: Water (0.1% FA)	44–120	n.p.	0.01–7.55	0.01–1.72	Liu et al., 2021
Fluoroquinolones, sulfonamides, β‐lactams, sulfonamides, β‐lactams, macrolides, tetracyclines, and phenicols	45	China	Urine	Oasis HLB SPE (60 mg, 3 mL, 30 µm)	LC–QqQ–MS	ZORBAX SB‐C18 (150 × 2.1 mm, 3.5 µm, Agilent)	MP A: Water (0.1% FA), MP B: ACN (0.1% FA)	74–112	n.p.	0.11–6.02	0.03–2.15	Liu et al., 2021
β‐lactams, linezolid, and β‐lactams inhibitors	10	Belgium	Plasma (healthy volunteer)	Protein precipitation	HRMS, ESI	Accucore phenyl hexyl column (100 × 2.1 mm, 2.6 µm, Thermo Fisher)	MP A: Water (0.01% FA), MP B: MeOH	87–97	1.0–5.0	250–1000	n.p.	Van Vooren & Verstraete, 2021
Sulfonamides, tetracyclines, and fluoroquinolones	30	China	Urine (male)	LLE	LC–QqQ–MS, ESI	Eclipse XDB‐C18 (150 × 2.1 mm, 3.5 µm)	MP A: Water (0.1% FA), MP B: MeOH (0.1% FA)	n.p.	n.p.	n.p.	n.p.	Zhou, Cuasquer, et al., 2021
Antibiotics	41	China	Urine (pregnant women)	HLB SPE	LC–QqQ–MS	ZORBAX SB‐C18 (150 × 2.1 mm, 3.5 µm)	MP A: Water (0.1% FA), MP B: ACN (0.1% FA)	n.p.	n.r.	n.p.	n.p.	Geng et al., 2021
Antibiotics	7	China	Plasma (healthy volunteer)	Protein precipitation	LC–PDA	ACQUITY UPLC HSS T3 column (50 × 2.1 mm, 1.8 µm)	MP A: Water (0.1% TFA), MP B: can	92–112	1.0–10.0	50–800	n.p.	Xie et al., 2022
Sulfonamides, fluoroquinolones, phenicols, macrolides, and tetracyclines	15	China	Urine	Enzymatic hydrolysis (β‐glucuronidase)	LC–QqQ–MS	Xbridge C18 (30 × 2.1 mm, 10 µm), UPLC HSS T3 (100 × 2.1, 1.8 µm)	Water (0.1% FA), MeOH (0.1% FA)	85–115	<15	n.p.	0.1–1	Zhang et al., 2022

Abbreviations: ACN, acetonitrile; ADI, acceptable daily intake; CSF, cerebrospinal fluid; EDTA, ethylenediaminetetraacetic acid; ESI, electrospray ionization; FA, formic acid; HLB, hydrophilic–lipophilic balance; HPLC, high‐performance liquid chromatography; HR‐MS, high‐resolution mass spectrometry; MP, mobile phase; n.p., not provided; ODS, octadecylsilyl; QQQ‐MS, triple quadrupole mass spectrometry; SPE, solid‐phase extraction; TDM, therapeutic drug monitoring.

## 2 SOURCES OF VETERINARY DRUG CONTAMINATION IN HUMAN, ANIMALS, FOOD, AND THE ENVIRONMENT

**FIGURE 2:**
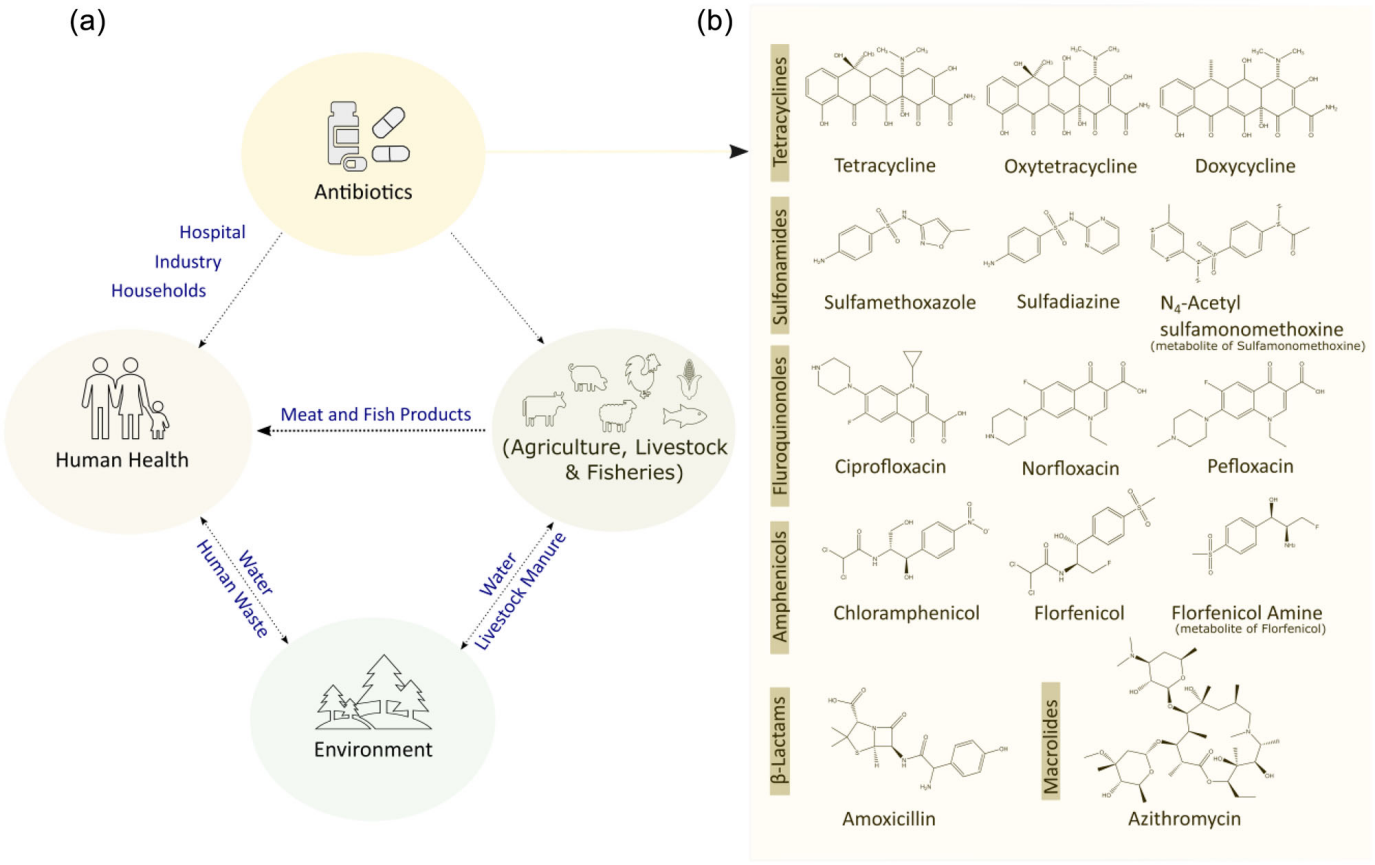
(a) Sources of unintended antibiotic contamination in humans, agriculture including livestock and fisheries, and the environment. (b) Major antibiotic groups and examples of commonly detected antibiotics and their metabolites in human biofluids.

Since the discovery and beginning of the indiscriminate use of antibiotics in human and veterinary medicine, antibiotics have been widely distributed in various parts of the environment (Hu et al., 2022). The major sources of residual antibiotics are wastewater from households, hospitals, the pharmaceutical industry, and livestock farms, and the improper disposal of wastewater and effluents represents an important source of residual antibiotics in the environment (Polianciuc et al., 2020). After medical use, substantial fractions of ingested antibiotics (30%–90%) are typically excreted as parent compounds, active metabolites, or conjugates via urine or feces, and therefore, household wastewater represents another important source of contamination for aquatic environments (Carvalho & Santos, 2016). Additionally, improper use of antibiotics in livestock farming can also lead to residual antibiotics in foods of animal origin (e.g., meat, milk, and egg) and contaminate the environment including soils, water, and crops through the use of manure as fertilizer and the irrigation of crops with wastewater (Polianciuc et al., 2020). Figure 2a summarizes potential sources of unintended residual antibiotics in humans, agriculture, livestock and fisheries, and the environment.

β‐Lactams (penicillin and cephalosporins), macrolides, fluoroquinolones, trimethoprim, and tetracyclines represent the most relevant group of antibiotics used for human applications (Huang et al., 2019). Besides human medical use, the livestock industry is a major consumer of several antibiotic groups including tetracyclines, β‐lactams, macrolides, and sulfonamides. Currently, 70–80 antibiotics and their metabolites can be detected in various animal‐derived foods, including meat (e.g., pork, chicken, and beef), milk, and aquatic products, and even in drinking water (Wang et al., 2017; Wang, Wang, Wang, Zhao, et al., 2016; Yamaguchi et al., 2015). Upon consumption, several emerging environmental pollutants exhibit both reversible and irreversible effects on human health, for example, due to their bioaccumulation or chronic toxicity (Di Poi et al., 2018; Liu et al., 2022). As emerging pollutants, antibiotics are widely used for medical treatment, for the prevention of infectious disease, and as a growth promotor, and chronic exposure may lead to various health risks. For instance, the long‐term administration of low doses of antibiotics can increase the risk of antibiotic‐resistant pathogenic strains and can alter the gut microbiome and further modify the bioavailability of dietary components and contaminants, such as pesticides (Zhan et al., 2018).

## 3 ASSESSMENT OF VA EXPOSURE IN HUMAN BIOFLUIDS

Biomarkers are measurable indicators of xenobiotic exposure, biological effects, or adverse health outcomes (Marín et al., 2018). Biomarkers of exposure can be parent compounds or metabolites that are present in human biofluids in free or conjugated forms (Yusa et al., 2015). In HBM studies, such biomarkers can be monitored in various tissues or biofluids and allow the evaluation of potential VA exposures, the prediction of exposure effects, and the development of policy‐level decisions and guidelines to protect human health.

Urine is often the sample matrix of choice for exposome studies due to the possibility of noninvasive sample collection, simple sample handling for large‐scale population studies, and large coverage of exogenous and endogenous metabolites (Cortejade et al., 2016). Urinary biomarkers provide short‐term exposure information, whereas plasma or serum concentrations represent long‐term exposure to antibiotics. Urine samples are typically collected over 24 h to ensure a realistic representation of the daily exposure and to minimize the temporal variation and variable urine concentration, which can partially be solved by adjusting the creatinine concentration. Alternatively, blood‐based samples are frequently used for HBM studies, although trained personnel are required for sampling. Blood plasma, in particular, has proven useful for the characterization of internal chemical exposure since it is in constant contact with human tissues and all organs (Yusa et al., 2015). Furthermore, exposure to various classes of VAs including sulfonamides, tetracyclines, fluoroquinolones, macrolides, and β‐lactams was also investigated by sampling other alternative sample matrices including cerebrospinal fluid (CSF), feces, or fingernails (Bellouard et al., 2020; Gómez‐Regalado et al., 2023; Wang et al., 2020). Upon uptake, antibiotics are metabolized via methylation, acetylation, or hydroxylation, and a considerable fraction is excreted in urine and feces as parent compounds or as conjugates. In this regard, feces represent an interesting and noninvasive alternative to monitoring residual antibiotics in human populations (Wang et al., 2020). Figure 2b shows a selection of the most relevant antibiotics for veterinary and human use and their metabolites that can be detected in human biofluids. To the best of our knowledge, these VAs were not studied in other noninvasive sample matrices such as hair, nails, meconium, or saliva in large‐scale HBM studies so far. In general, the choice of a suitable biospecimen for HBM studies depends on the specific exposure and the target population, but the simultaneous investigation of various biofluids may allow a more comprehensive picture of the total exposure.

## 4 ANALYTICAL METHODS FOR HUMAN BIOMONITORING OF VAs

Analyzing trace amounts of VAs in biological samples is a nontrivial task, and analytical workflows include many steps including sampling, protein precipitation, extraction, cleanup, and, finally, analyte separation and detection. A full summary of HBM studies investigating antibiotic exposure, including the extraction and cleanup steps used, analyte separation, detection methods, and analytical performance for multiple biofluids, is presented in Table 1, and single steps of the analytical pipeline are discussed in the following section.

### 4.1 Sample (pre)treatment

Suitable extraction and sample (pre)treatment methods are prerequisites for the analysis of xenobiotics in complex biological matrices. Typically, such methods include analyte extraction and purification steps, but further cleanup or concentration steps might be required to improve method sensitivity and selectivity (Gu et al., 2023). Most reported protocols include extraction methods based on protein precipitation/liquid extraction complemented by filtration or acidification to remove interfering substances or to adjust pH values to optimize extraction efficiency for target analytes. Furthermore, more sophisticated techniques have been used in recent HBM studies including SPE (solid‐phase extraction), dispersive solid‐phase extraction (d‐SPE), SPME (solid‐phase microextraction), and dispersive liquid–liquid microextraction (DLLME) (Bellouard et al., 2020; Jourdil et al., 2013; Legrand et al., 2016; Sime et al., 2014; Stajic et al., 2018; Van Vooren & Verstraete, 2021). Hydrolysis of conjugates, for example, by incubation with β‐glucuronidase, can be applied before extraction to analyze the sum of free antibiotics and their conjugated forms (Hu et al., 2022), and the efficiency of such enzymatic hydrolysis assays can be monitored, for example, using 4‐methylumbelliferone glucuronide (Wang et al., 2014). However, it is noteworthy that enzymatic treatment (e.g., using β‐glucuronidase) is an additional possible source of sample contamination with various xenobiotic compounds that needs to be evaluated in detail (Fareed et al., 2022).

During method development, various experimental conditions including solvents, the ratio of sample extraction solvents, pH values, the addition of salts, complexing agents (e.g., Na_2_EDTA), or other modifiers, as well as the choice of reconstituting solvents need to be optimized (Peris‐Vicente et al., 2022). For example, Na_2_EDTA can be used to complex matrix cations and reduce their interaction with antibiotics such as tetracyclines (Jin et al., 2010). Sample and solvent pH values can affect the stability of antibiotics in a solution as well as the ionization efficiencies when using LC–MS for analyte detection. For instance, several antibiotics such as β‐lactam antibiotics (e.g., amoxicillin and ampicillin), tetracyclines, and sulfonamides, as well as antibiotics containing aglycone rings (e.g., azithromycin, clarithromycin, and roxithromycin), show improved stability under acidic conditions, while other VAs such as macrolides with p*K*_a_ values between 7.1 and 9.9 are more stable in basic pH conditions (Hu et al., 2022; Huang et al., 2019).

In HBM studies, the complexity of the sample matrices can jeopardize the performance of straightforward sample preparation methods, such as protein precipitation. As such, they are not always suitable to meet the sensitivity requirements of ultratrace analysis, and various techniques have been developed to remove interfering matrix components prior to analyte detection. In this regard, SPE using hydrophobic–lipophilic balance (HLB) adsorbent has become one popular sample cleanup technique for the extraction of VAs from human biofluids (Geng et al., 2021; Huang et al., 2019; Liu et al., 2022; Wang et al., 2021; Wang, Tang, et al., 2018; Zhu et al., 2020). Different types of sorbents are suitable to tailor extraction procedures. For example, HLB sorbents enable the retention of VAs and cover a wide range of polarities (polar to nonpolar), p*K*_a_ values, and molecular weights (Geng et al., 2020; Hu et al., 2022; Jin et al., 2010; Wang et al., 2014; Wang, Wang, Wang, Fang, et al., 2016). Additionally, different types of mixed‐mode SPE, such as reversed‐phase and anion‐exchange MAX sorbent or MCX sorbents, are used to extract amphenicol antibiotics (e.g., chloramphenicol, thiamphenicol, and florfenicol) from urine (Yao et al., 2018) or to extract β‐lactam antibiotics and can cover additional analytes within a wide range of physicochemical properties (Colin et al., 2013). However, a careful choice of the sorbent material is crucial, as the selection can substantially influence the results of the applied extraction approach. For instance, MCX sorbents were reported to outperform MAX, WAX, and WCX sorbents for the analysis of β‐lactams with overall extraction efficiencies of 73%–96%. For quinolone antibiotics, prime HLB adsorbent was used to remove common matrix interference (∼95%) including proteins, phospholipids, and salts in urine and serum (Hu et al., 2022; Wang et al., 2019). In addition to the choice of the sorbent, careful optimization of the full extraction workflow (e.g., the used elution solvents) is necessary to optimize the extraction efficiency. For instance, Yao et al. (2018) chose ethyl acetate over methanol or acetonitrile to elute amphenicols in urine. Furthermore, d‐SPE was applied to extract 18 antibiotics of five different classes with considerable efficiency (73%–136%) (Yao et al., 2018).

As alternatives to SPE‐based methods, other extraction and purification methods, including LLE and DLLME, are widely used to extract antibiotics from human biofluids (Ferrone et al., 2018; Jia et al., 2013; Minohara et al., 2021; Zhou, Cuasquer, et al., 2021). For instance, Jia et al. (2013) used DLLME based on the solidification of floating organic droplets for the extraction of five macrolide antibiotics (azithromycin, erythromycin, roxithromycin, clarithromycin, and dirithromycin) in urine with high extraction efficiencies between 89% and 95%. Moreover, molecularly imprinted polymer‐coated hollow fibers (MIP‐HFs) in combination with SPME were used by Barahona et al. (2019) to extract fluoroquinolone antibiotics from urine samples. This approach allows to combine the benefits of selective recognition of target analytes, low‐solvent consumption, and on‐site testing. Due to the selectivity of HFs for single analytes, application for larger analyte panels may be challenging and can result in unsatisfactory repeatability (9.5%–24%), for example, as shown for the extraction of quinolone antibiotics from urine samples (Barahona et al., 2019).

### 4.2 Analyte separation and detection

Depending on the scope of the study, various methods including microbiological, immunological, and instrumental analytical methods are used for the detection of antibiotics in biofluids (Huang et al., 2019). However, microbiological assays are time‐consuming and provide only low sensitivity and specificity, whereas immunoassays (UV spectrophotometry, spectrofluorometry, and immunofluorescence) require tedious manual operation and are not suitable for large‐scale trace analysis (Hu et al., 2022). Notably, these assays (immunoassay and electrochemical) showed lower sensitivity, with limits of detection ranging from 0.003 to 510 ng/mL, compared to advanced (tandem) MS, which had limits of detection from 0.002 to 14.3 ng/mL (Hu et al., 2022), and are typically not suitable for exposome‐scale trace analysis. Therefore, instrumental analytical methods taking advantage of modern MS typically provide a suitable toolbox for the determination of antibiotics in biological matrices including urine, plasma, serum, or whole blood (Pauter et al., 2020) and represent the typically used platforms for exposome‐scale HBM methods (Hossain et al., 2024; Krausová et al., 2023; Vitale et al., 2021).

Due to the high complexity of biological sample matrices, analyte separation prior to detection is necessary, and liquid chromatography (LC) is typically the method of choice to separate VAs. Reversed‐phase columns with various column chemistries can be used for the chromatographic separation of VAs and include Zorbax SB‐C18, BEH‐C18, HSS T3, Xbridge C18, or Gemini C18 columns (see Table 1). Especially, Zorbax SB‐C18 columns provide stable column chemistry and can be used even with high column temperatures (<80°C) or low pH values (<1) and were reportedly used for the rapid separation of six antibiotic classes (Li et al., 2017; Liu et al., 2020; Wang et al., 2019). BEH‐C18 columns are considered a universal column choice, as they provide the widest usable pH range (pH 1–12) and were reportedly used to analyze 10 antibiotic classes (Abdulla et al., 2017; Carlier et al., 2015; Jin et al., 2010; Wang et al., 2020; Yao et al., 2018). However, Jin et al. (2010) reported that BEH‐C18 may only weakly interact with highly polar analytes. In contrast, HSS T3 columns were reported to offer better retention of highly polar compounds and were used to determine 18 antibiotics from five different classes (Wang, Yang, et al., 2018). In addition to the choice of the chromatographic column, mobile phase compositions, chromatographic gradients, and flow rates influence the separation efficiency. Besides various combinations of organic phases, typically methanol and/or acetonitrile, and an aqueous phase (water), mobile phase modifiers including formic acid, ammonium acetate/formate, or ammonium fluoride can be used to enhance ionization efficiency, improve peak shape, and ensure an efficient and reliable analyte separation (Geng et al., 2021; Hossain et al., 2024; Minohara et al., 2021; Wang et al., 2020; Zhang et al., 2022).

Various detector types were successfully used for the quantification of VAs in combination with LC, including spectral detectors such as ultraviolet (UV) (Denooz & Charlier, 2008; Legrand et al., 2016; McWhinney et al., 2010), photodiode array (PDA) (Briscoe et al., 2012; Ferrone et al., 2018; Xie et al., 2022), and diode‐array detector (DAD)–fluorescence detector, which allow the detection and quantification of compounds based on their characteristic spectra (Fernandez‐Torres et al., 2010). However, such approaches may suffer from limited specificity, because various analytes including VAs can absorb or emit light of the same wavelength (e.g., cefazolin and ofloxacin), which hampers simultaneous analysis using LC–UV (Legrand et al., 2016).

In addition, these LC methods have a relatively longer analysis time and low sensitivity compared to LC–MS. Triple quadruple MS instruments (LC–QqQ–MS/MS) represent the most widely used class of mass detectors used for the quantitative analysis of antibiotics in biofluids. Applications using multiple reaction monitoring (MRM) allow trace analysis with high sensitivity and specificity for multiple analytes in a single run (Bellouard et al., 2020; Colin et al., 2013; Geng et al., 2021; Huang et al., 2020; Kiriazopoulos et al., 2017; Liu et al., 2017, 2021; Yao et al., 2018; Zhang et al., 2020). LC–MS/MS provides precise quantification and identification of analytes in complex biological matrices while minimizing interferences from co‐eluting analytes. Traditionally, biomonitoring of VAs was limited to a single group of VAs such as β‐lactams (Carlier et al., 2012; Colin et al., 2013; Sime et al., 2014), tetracyclines (Jin et al., 2010), fluoroquinolones (Canada‐Canada et al., 2007), macrolides (Jia et al., 2013), and aminoglycosides (Lu & Feng, 2007), with each group containing <15 compounds. With the advent of advanced MS and comprehensive sample preparation techniques in recent years, major groups of antibiotics such as β‐lactams, sulfonamides, tetracyclines, fluoroquinolones, macrolides, and amphenicols can now be analyzed simultaneously in urine with up to 45 different VAs per run (Geng et al., 2020; Liu et al., 2021; Wang et al., 2021; Wang, Tang, et al., 2018; Zhang et al., 2021; Zhou, Cuasquer, et al., 2021; Zhou, Zhu, et al., 2021). Furthermore, multianalyte screening and multiresidue quantification methods were successfully developed and applied to analyze multiple groups of environmentally relevant chemicals, including VAs, pesticides, or parabens, for the evaluation of human exposition (Cortejade et al., 2016; Hossain et al., 2024).

Recent instrumental advances and related improvements in detector sensitivity led to a rising number of applications of HRMS, including quadrupole‐time‐of‐flight (QToF) and Orbitrap detectors (Marín et al., 2018; Oesterle et al., 2021; Van Vooren & Verstraete, 2021). In comparison to targeted LC–MS/MS, such approaches can be used for nontargeted analysis (NTA) and suspect and targeted screening in full scan mode. High mass resolution and mass accuracy, combined with the possibility to acquire precursor (MS^1^) and fragment spectra (MS^2^) in a single analytical run, make HRMS(/MS) suitable for compound discovery as well as qualitative and quantitative analysis (Cortejade et al., 2016; Oesterle et al., 2021; Van Vooren & Verstraete, 2021). The utilization of HRMS‐based assays and more advanced targeted multianalyte, next‐generation HBM approaches for large‐scale HBM studies shows promising results. Because of its high mass resolution and accuracy capabilities for the detection of unknown compounds and molecular characterization, HRMS is particularly valuable in metabolomics studies. LC–MS/MS and LC‐HR/MS(/MS)) approaches offer greater potential with superior specificity, sensitivity, and comprehensive multianalyte data‐output capabilities over other methods for in‐depth HBM studies. Such approaches will allow to further increase the coverage of VAs and their metabolites in a single assay and offer an elegant opportunity to integrate VA analysis into the analytical exposomics framework.

## 5 REAL‐LIFE EXPOSURE SCENARIOS IN HUMAN BIOFLUIDS

Collecting real‐life exposure data for multiple classes of antibiotics in parallel is essential for a successful exposure and risk assessment for human populations including vulnerable groups. Nevertheless, the number and coverage of published HBM studies monitoring human exposure to multiple classes of antibiotics are still limited (see Table 2). Interestingly, most of the published literature reports on VAs in the last couple of years present exposure data from Asia and especially from China (Table 1). In recent HBM studies, VAs were most frequently detected in human urine and plasma, but other sample matrices including feces (Wang et al., 2020), CSF (Bellouard et al., 2020), and breast milk (Kiriazopoulos et al., 2017) were also investigated. In these studies, fluoroquinolones, sulfonamides, and tetracyclines were the antibiotic groups with the highest detectin frequencies, followed by β‐lactams, amphenicols, and macrolides (Table 3). Fluoroquinolone antibiotics were most frequently detected in urine samples with individual detection frequencies ranging from 19% to 94% (median 33%), whereas sulfonamides, tetracyclines, and macrolides were detected in 3.6%–79%, 3.7%–48%, and 2.1%–30% of the analyzed samples with median detection frequencies of 34%, 31%, and 13%, respectively. The detection frequency for amphenicols ranged from 1.6% to 47%, with a median of 20%. Observed antibiotic concentrations (95% confidence interval [CI]) spanned a wide concentration range, with reported urine levels between 0.05 and 55 ng/mL (median: 0.81 ng/mL) for fluoroquinolones, 0.038 and 24 ng/mL (median: 0.75 ng/mL) for sulfonamides, 0.14 and 105 ng/mL (1.26 ng/mL) for tetracyclines, 0.03 and 183 ng/mL (median: 1.07 ng/mL) for macrolides, 0.98 and 19.5 ng/mL (median: 2.6 ng/mL) for β‐lactams, and 0.020 and 22.83 ng/mL (median: 0.32 ng/mL) for amphenicols (Table 2). The highest antibiotic concentrations were observed for macrolide antibiotics, followed by fluoroquinolones and tetracyclines. Reported urinary concentrations of individual antibiotics (ng/mL) are summarized in Table 2 and include data for various sub‐populations (children, pregnant women, adults, and elderly).

**TABLE 2:** Article information and reported veterinary drugs/antibiotics concentration (ng/mL) in human biofluids from human biomonitoring studies.

Article information	1	2	3	4	5	6	7	8	9	10	11	12	13	14	15	16	17	18	19	20	21	22	23	24	25
Population type	A	A	C	PW	C	PW	C	C	C	PW	C	PW^a^	GP	EA	GP	C	A	EA	PW	C	C	A	EA	A	A
Biofluid samples	Urine	Urine	Urine	Urine	Urine	Urine	Urine	Urine	Urine	Urine	Urine	Urine	Urine	Urine	Feces	Urine	Urine	Urine	Urine	Urine	Urine	Urine	Urine	Urine	Serum
LC‐MS method	LC–MS/MS	LC–Q‐ToF	LC–Q‐ToF	LC–MS/MS	LC–Q‐ToF	LC–MS/MS	LC–MS/MS	LC–Q‐ToF	LC–MS/MS	LC–MS/MS	LC–MS/MS	LC–MS/MS	LC–MS/MS	LC–MS/MS	LC–MS/MS	LC–MS/MS	LC–MS/MS	LC–MS/MS	LC–MS/MS	LC–MS/MS	LC–MS/MS	LC–MS/MS	LC–MS/MS	LC–MS/MS	LC–MS/MS
Sample size (*n*)	30	822	284	9136	1134	762	31	393	107	126	735	2287	87	713	180	326	263	102	302	249	6	12	990	177	107

Groups	Individual Antibiotics (in ng/mL)	1	2	3	4	5	6	7	8	9	10	11	12	13	14	15	16	17	18	19	20	21	22	23	24	25
Sulfonamides	Sulfamethoxazole	0.84–2.9	n.d.	n.d.	0.11	n.d.	0.16	0.03	0.3	n.i.	n.i.	0.021	n.d.	2.82	n.d.	45.4	n.d.	n.d.	n.d.	0.2	n.i.	n.i.	n.i.	n.d.	n.d.	n.d.
	Acetylated sulfamethoxazole	n.i.	n.i.	n.i.	n.i.	n.d.	n.i.	n.i.	n.i.	n.i.	n.i.	n.i.	n.i.	n.i.	n.i.	n.i.	n.i.	n.i.	n.i.	n.i.	n.i.	n.i.	n.i.	n.i.	n.i.	n.i.
	Sulfaclozine	n.i.	n.i.	n.d.	n.d.	n.i.	n.i.	n.i.	n.i.	n.i.	n.i.	n.i.	n.d.	n.i.	17.87	n.i.	10.2	14.8	11.4	n.d.	n.i.	n.i.	n.i.	23.7	n.i.	n.i.
	Trimethoprim	n.i.	0.46	0.75	1.89	0.48	0.25	n.i.	0.5	n.i.	n.i.	n.d.	2.08	1.4	1.76	38.3	0.6	1.6	4.3	1.4	n.i.	26.1	n.i.	1.4	78–667	1.9
	Sulfamethazine	n.d.	0.28	n.d.	0.17	n.d.	n.d.	n.i.	5.3	n.i.	n.i.	n.d.	0.19	2.02	n.d.	41.7	n.d.	n.d.	n.d.	n.d.	n.d.	n.d.	n.i.	n.d.	n.i.	n.d.
	Acetylated sulfamethazine	n.i.	n.i.	n.d.	n.i.	n.d.	n.i.	n.i.	n.i.	n.i.	n.i.	n.i.	n.i.	n.i.	n.i.	n.i.	n.i.	n.i.	n.i.	n.i.	n.i.	n.i.	n.i.	n.i.	n.i.	n.i.
	Sulfadiazine	n.d.	n.d.	n.d.	n.d.	n.d.	0.04	n.i.	n.d.	n.i.	n.i.	n.d.	n.d.	0.26	n.d.	45.5	n.d.	n.d.	n.d.	n.d.	n.d.	49.9	n.i.	n.d.	n.i.	n.d.
	Acetylated sulfadiazine	n.i.	n.i.	n.d.	n.i.	n.d.	n.i.	n.i.	n.i.	n.i.	n.i.	n.i.	n.i.	n.i.	n.i.	n.i.	n.i.	n.i.	n.i.	n.i.	n.i.	n.i.	n.i.	n.i.	n.i.	n.i.
	Sulfadimethylpyrimidine	0.25–0.29	n.i.	n.i.	n.i.	n.i.	n.i.	n.i.	n.i.	n.i.	n.i.	n.i.	n.i.	n.i.	n.i.	n.i.	n.i.	n.i.	n.i.	n.i.	n.i.	n.i.	n.i.	n.i.	n.i.	n.i.
	Sulfachloropyridazine	n.d.	n.i.	n.i.	n.d.	n.i.	n.i.	n.i.	n.i.	n.i.	n.i.	n.i.	n.d.	n.i.	n.d.	26.1	n.i.	n.i.	n.i.	n.d.	n.i.	n.i.	n.i.	n.d.	n.i.	n.i.
	Sulfamonomethoxine	n.d.	n.i.	n.i.	0.39	n.i.	n.i.	n.i.	n.i.	n.i.	n.i.	n.i.	n.d.	n.i.	1.05	n.i.	n.d.	n.d.	n.d.	n.d.	n.i.	n.i.	n.i.	0.8	n.i.	n.i.
	Sulfadimethoxine	n.d.	n.i.	n.i.	n.i.	n.i.	n.i.	0.04	n.i.	n.i.	n.i.	n.i.	n.i.	n.i.	n.i.	16.8	n.i.	n.i.	n.i.	n.i.	n.i.	n.i.	n.i.	n.i.	n.i.	8.8
	Sulfamerazine	n.i.	n.i.	n.i.	n.i.	n.i.	n.i.	0.05	n.i.	n.i.	n.i.	n.i.	n.i.	n.i.	n.i.	n.i.	n.i.	n.i.	n.i.	n.i.	n.i.	n.i.	n.i.	n.i.	n.i.	4.6
	Sulfaquinoxaline	n.i.	n.i.	n.i.	n.i.	n.d.	n.i.	n.i.	n.i.	n.i.	n.i.	n.i.	n.i.	8.83	n.i.	n.i.	n.i.	n.i.	n.i.	n.i.	n.i.	n.i.	n.i.	n.i.	n.i.	n.i.
	Sulfapyridine	n.i.	n.i.	n.i.	n.i.	n.i.	n.i.	n.i.	n.i.	n.i.	n.i.	n.i.	n.i.	n.i.	n.i.	n.i.	n.i.	n.i.	n.i.	n.i.	n.i.	n.i.	n.i.	n.i.	n.i.	n.d.
	Sulfaguanidine	n.d.	n.i.	n.i.	n.i.	n.i.	n.i.	n.i.	n.i.	n.i.	n.i.	n.i.	n.i.	n.i.	n.i.	n.i.	n.i.	n.i.	n.i.	n.i.	n.i.	n.i.	n.i.	n.i.	n.i.	n.i.
	Sulfaphenazole	n.d.	n.i.	n.i.	n.i.	n.i.	n.i.	n.i.	n.i.	n.i.	n.i.	n.i.	n.i.	n.i.	n.i.	n.i.	n.i.	n.i.	n.i.	n.i.	n.i.	n.i.	n.i.	n.i.	n.i.	n.i.
	Sodium sulfacetamide	n.d.	n.i.	n.i.	n.i.	n.i.	n.i.	n.i.	n.i.	n.i.	n.i.	n.i.	n.i.	n.i.	n.i.	n.i.	n.i.	n.i.	n.i.	n.i.	n.i.	n.i.	n.i.	n.i.	n.i.	n.i.
	Oxolinic acid	n.d.	n.i.	n.i.	n.i.	n.i.	n.i.	n.i.	n.i.	n.i.	n.i.	n.i.	n.i.	n.i.	n.i.	n.i.	n.i.	n.i.	n.i.	n.i.	n.i.	n.i.	n.i.	n.i.	n.i.	n.i.
	Sulfameter	n.i.	n.i.	n.i.	n.d.	n.i.	n.i.	n.i.	n.i.	n.i.	n.i.	n.i.	n.d.	n.i.	n.i.	n.i.	n.i.	n.i.	n.i.	n.i.	n.i.	n.i.	n.i.	n.i.	n.i.	n.i.
	Sulfachinoxaline	n.i.	n.i.	n.i.	n.d.	n.i.	n.i.	n.i.	n.i.	n.i.	n.i.	n.i.	n.d.	n.i.	n.i.	n.i.	n.d.	n.d.	n.d.	n.d.	n.i.	n.i.	n.i.	n.i.	n.i.	n.i.
	Sulfathiazole	n.i.	n.i.	n.i.	n.i.	n.i.	n.i.	n.i.	n.i.	n.i.	n.i.	n.i.	n.i.	n.i.	n.i.	n.i.	n.i.	n.i.	n.i.	n.i.	n.i.	n.i.	n.i.	n.i.	n.i.	n.d.
	Sulfamethoxypyridazine	n.d.	n.i.	n.i.	n.i.	n.i.	n.i.	n.i.	n.i.	n.i.	n.i.	n.i.	n.i.	n.i.	n.i.	n.i.	n.i.	n.i.	n.i.	n.i.	n.i.	n.i.	n.i.	n.i.	n.i.	n.i.
Macrolides	Erythromycin	n.i.	n.i.	n.i.	0.60	n.i.	n.d.	0.19	n.d.	n.i.	n.i.	n.d.	n.d.	n.i.	1.2	36.8	1.4	n.d.	n.d.	0.2	78.1	10.8	n.i.	1.2	n.d.	n.d.
	Dehydrated erythromycin	n.i.	n.i.	n.i.	n.i.	n.d.	n.i.	n.i.	n.i.	n.i.	n.i.	n.i.	n.i.	n.i.	n.i.	n.i.	n.i.	n.i.	n.i.	n.i.	n.i.	n.i.	n.i.	n.i.	n.i.	n.i.
	Clarithromycin	n.i.	n.d.	n.d.	n.d.	n.d.	n.d.	n.i.	n.d.	n.i.	n.i.	n.d.	n.d.	n.d.	n.d.	n.i.	n.d.	n.d.	n.d.	n.d.	183	n.d.	n.i.	n.d.	n.i.	n.d.
	Azithromycin	n.i.	n.d.	9.87	0.40	1.5	0.07	n.i.	51.9	n.i.	n.i.	n.d.	n.d.	0.98	0.23	n.i.	2.2	28.4	82.3	0.03	62.1	142.7	n.i.	0.2	n.i.	n.d.
	Roxithromycin	n.i.	n.d.	n.d.	n.d.	n.d.	n.i.	n.i.	n.d.	n.i.	n.i.	n.i.	n.d.	n.d.	n.d.	34.7	0.3	2.0	1.2	n.d.	n.d.	n.d.	n.i.	n.d.	n.i.	n.d.
	Tylosin	n.i.	n.i.	n.i.	n.d.	n.d.	n.i.	n.i.	n.d.	n.i.	n.i.	n.i.	n.i.	n.i.	n.i.	23.7	n.i.	n.i.	n.i.	1.4	n.i.	n.d.	n.i.	n.i.	n.i.	n.i.
	Tilmicosin	n.i.	n.i.	n.i.	n.i.	n.i.	n.i.	n.i.	n.i.	n.i.	n.i.	n.i.	n.d.	n.i.	n.i.	n.i.	n.i.	n.i.	n.i.	n.d.	n.i.	n.i.	n.i.	n.i.	n.i.	n.i.
β‐Lactams	Penicillin G	n.i.	n.i.	n.i.	19.50	n.i.	n.i.	n.i.	n.i.	n.i.	n.i.	n.i.	n.i.	n.i.	n.i.	n.i.	n.i.	n.i.	n.i.	n.d.	n.i.	n.i.	n.i.	n.i.	n.d.	n.i.
	Penicillin V	n.i.	n.i.	n.i.	5.73	n.i.	n.i.	n.i.	n.i.	n.i.	n.i.	n.i.	n.i.	n.i.	2.39	n.i.	n.d.	0.9	0.7	2.0	n.i.	n.i.	n.i.	2.8	n.i.	n.i.
	Amoxicillin	n.i.	n.i.	n.i.	n.d.	n.i.	n.i.	n.i.	n.i.	n.i.	n.i.	n.i.	n.i.	n.i.	1.02	43.7	11.7	1.4	n.d.	n.d.	15.7	n.d.	n.i.	1.0	22.7	n.i.
	Ampicillin	n.i.	n.i.	n.i.	n.i.	n.i.	n.i.	n.i.	n.d.	n.i.	n.i.	n.i.	n.i.	n.i.	n.i.	49.5	n.i.	n.i.	n.i.	n.i.	n.i.	n.i.	n.i.	n.i.	19.6–683	n.i.
	Cephalexin	n.i.	n.i.	n.i.	n.i.	n.i.	n.i.	n.i.	n.i.	n.i.	n.i.	n.i.	n.i.	n.i.	n.i.	49.4	n.i.	n.i.	n.i.	n.i.	n.i.	n.i.	n.i.	n.i.	n.i.	n.i.
	Cefoperazone	n.i.	n.i.	n.i.	n.i.	n.i.	n.i.	n.i.	n.i.	n.i.	n.i.	n.i.	n.i.	n.i.	n.i.	n.i.	n.i.	n.i.	n.i.	n.i.	n.i.	n.i.	n.i.	n.i.	n.i.	7.6
	Ceftiofur	n.i.	n.i.	n.i.	n.d.	n.i.	n.i.	n.i.	n.i.	n.i.	n.i.	n.i.	n.d.	n.i.	n.i.	n.i.	n.i.	n.i.	n.i.	n.i.	n.i.	n.i.	n.i.	n.i.	n.i.	n.i.
	Cefquinome	n.i.	n.i.	n.i.	n.d.	n.i.	n.i.	n.i.	n.i.	n.i.	n.i.	n.i.	n.d.	n.i.	n.i.	n.i.	n.i.	n.i.	n.i.	n.d.	n.i.	n.i.	n.i.	n.i.	n.i.	n.i.
	Cefaclor	n.i.	n.i.	n.i.	n.d.	n.i.	n.i.	n.i.	n.d.	n.i.	n.i.	n.i.	n.d.	n.i.	n.d.	n.i.	n.d.	n.d.	n.d.	n.i.	n.i.	n.i.	n.i.	n.d.	n.i.	n.d.
	Cefotaxime	n.i.	n.i.	n.i.	n.d.	n.i.	n.i.	n.i.	n.i.	n.i.	n.i.	n.i.	n.d.	n.i.	n.d.	n.i.	n.d.	n.d.	n.d.	n.d.	n.i.	n.i.	n.i.	n.d.	n.i.	n.i.
	Clavulanic acid	n.i.	n.i.	n.i.	n.i.	n.i.	n.i.	n.i.	n.i.	n.i.	n.i.	n.i.	n.i.	n.i.	n.i.	n.i.	n.i.	n.i.	n.i.	n.i.	n.i.	n.i.	n.i.	n.i.	n.d.	n.i.
	Cefuroxime	n.i.	n.i.	n.i.	n.i.	n.i.	n.i.	n.i.	n.i.	n.i.	n.i.	n.i.	n.i.	n.i.	n.i.	n.i.	n.i.	n.i.	n.i.	n.i.	n.i.	n.i.	n.i.	n.i.	n.d.	n.i.
	Ceftriaxone	n.i.	n.i.	n.i.	n.i.	n.i.	n.i.	n.i.	n.i.	n.i.	n.i.	n.i.	n.i.	n.i.	n.i.	n.i.	n.i.	n.i.	n.i.	n.i.	n.i.	n.i.	n.i.	n.i.	n.d.	n.i.
	Cefoperazone	n.i.	n.i.	n.i.	n.i.	n.i.	n.i.	n.i.	n.i.	n.i.	n.i.	n.i.	n.i.	n.i.	n.i.	n.i.	n.i.	n.i.	n.i.	n.i.	n.i.	n.i.	n.i.	n.i.	n.i.	n.d.
	Penicillin	n.i.	n.i.	n.i.	n.i.	n.i.	n.i.	0.13	n.i.	n.i.	n.i.	n.i.	n.i.	n.i.	n.i.	n.i.	n.i.	n.i.	n.i.	n.i.	n.d.	n.d.	n.i.	n.i.	n.i.	n.d.
Tetracyclines	Oxytetracycline	0.94–41.2	n.d.	n.d.	0.48	n.d.	n.i.	n.d.	n.d.	0.5	0.37	n.i.	0.46	2.07	1.28	38.7	28.5	7.1	9.1	0.6	40.3	n.i.	n.i.	1.9	n.i.	n.d.
	Chlorotetracycline	1.2–3.7	n.d.	0.31	0.19	0.66	n.i.	n.i.	n.d.	0.96	0.50	n.i.	n.d.	1.07	0.98	36.6	n.d.	n.d.	n.d.	0.4	n.i.	n.d.	n.i.	1.3	n.i.	n.i.
	Tetracycline	1.1–2.0	n.d.	n.d.	0.41	n.d.	1.68	n.d.	n.d.	0.14	0.46	n.d.	0.45	70.49	2.11	n.i.	n.d.	12.3	4.5	0.4	105	n.d.	n.i.	2.2	n.d.	n.d.
	Terramycin	n.i.	n.i.	n.i.	n.i.	n.i.	n.i.	n.i.	n.i.	n.i.	n.i.	n.i.	n.i.	n.i.	n.i.	n.i.	n.i.	n.i.	n.i.	n.i.	n.i.	n.d.	n.i.	n.i.	n.i.	n.i.
	Doxycycline	5.9–23.7	n.d.	n.d.	1.81	4.4	n.i.	0.05	n.i.	4.69	1.54	n.i.	2.1	12.55	1.58	39.4	n.d.	2.2	n.d.	2.4	n.i.	n.i.	n.i.	1.9	n.i.	n.d.
Fluroquinolones	Pefloxacin	1.2–2.8	n.i.	n.d.	0.17	n.i.	n.i.	n.i.	n.i.	n.i.	n.i.	n.i.	n.d.	n.i.	n.d.	n.i.	n.d.	n.d.	n.d.	0.2	n.i.	n.i.	n.d.	n.d.	n.i.	n.i.
	Lomefloxacin	1.0–15.4	n.i.	n.d.	n.d.	n.i.	n.i.	n.i.	n.i.	n.i.	n.i.	n.i.	n.d.	n.i.	n.d.	n.i.	n.d.	n.d.	n.d.	0.03	n.i.	n.i.	n.i.	n.d.	n.i.	n.i.
	Danofloxacin	1.1–16.4	n.i.	n.i.	n.d.	n.d.	n.i.	n.i.	n.i.	n.i.	n.i.	n.i.	n.d.	n.i.	n.d.	n.i.	n.d.	n.d.	n.d.	n.d.	n.i.	n.i.	n.d.	n.d.	n.i.	n.i.
	Sarafloxacin	3.0–239	n.i.	n.i.	0.05	n.i.	n.i.	n.i.	n.i.	n.i.	n.i.	n.i.	n.d.	n.i.	n.d.	n.i.	n.d.	n.d.	n.d.	n.d.	n.i.	n.i.	n.d.	n.d.	n.i.	n.i.
	Ofloxacin	1.6–163.1	2.85	0.88	0.50	0.29	0.35	n.i.	1.2	n.i.	n.i.	n.d.	0.6	10.12	4.09	35.4	1.9	3.7	4.5	0.5	54.7	n.d.	n.i.	3.7	n.i.	33.3
	Difloxacin	0.22–2.5	n.d.	n.i.	n.d.	n.i.	n.i.	n.i.	n.i.	n.i.	n.i.	n.i.	n.d.	n.i.	n.d.	n.i.	n.d.	n.d.	n.d.	n.d.	n.i.	n.i.	n.d.	n.d.	n.i.	n.i.
	Enrofloxacin	1.1–3.4	n.d.	n.d.	0.34	2.8	0.08	0.04	0.2	n.i.	n.i.	0.016	0.35	2.78	0.63	29.2	n.d.	1.6	n.d.	0.2	n.d.	n.d.	n.i.	0.7	n.i.	4.9
	Ciprofloxacin	4.7–21.3	3.93	3.89	2.43	3.2	0.59	0.10	0.9	n.i.	n.i.	0.073	2.92	n.i.	4.16	37.5	1.9	7.1	2.9	2.8	23.8	n.d.	n.d.	4.4	26–847	3.9
	Norfloxacin	4.2–24.0	n.d.	n.d.	0.14	n.d.	0.44	0.04	0.3	n.i.	n.i.	0.056	n.d.	n.i.	3.88	n.i.	1.6	2.0	1.8	0.2	n.d.	n.d.	n.d.	4.5	n.i.	6.4
	Orbifloxacin	0.06–1.0	n.i.	n.i.	n.i.	n.i.	n.i.	n.i.	n.i.	n.i.	n.i.	n.i.	n.i.	n.i.	n.i.	n.i.	n.i.	n.i.	n.i.	n.i.	n.i.	n.i.	n.i.	n.i.	n.i.	n.i.
	Fleroxacin	0.24–1.7	n.i.	n.i.	n.i.	n.i.	n.i.	n.i.	n.i.	n.i.	n.i.	n.i.	n.i.	n.i.	n.i.	n.i.	n.i.	n.i.	n.i.	n.i.	n.i.	n.i.	n.i.	n.i.	n.i.	n.i.
	Levofloxacin	n.i.	n.i.	n.i.	n.i.	n.i.	n.i.	n.i.	n.i.	n.i.	n.i.	n.i.	n.i.	n.i.	n.d.	n.i.	n.d.	n.d.	n.d.	n.i.	n.i.	n.i.	n.i.	n.d.	n.i.	n.i.
	Sparfloxacin	n.d.	n.i.	n.i.	n.i.	n.i.	n.i.	n.i.	n.i.	n.i.	n.i.	n.i.	n.i.	n.i.	n.i.	n.i.	n.i.	n.i.	n.i.	n.i.	n.i.	n.i.	n.i.	n.i.	n.i.	n.i.
	Flumequine	n.i.	n.i.	n.i.	n.i.	n.i.	n.i.	n.i.	n.i.	n.i.	n.i.	n.i.	n.i.	n.i.	n.i.	n.i.	n.i.	n.i.	n.i.	n.i.	n.i.	n.i.	n.d.	n.i.	n.i.	n.i.
	Enoxacin	n.i.	n.i.	n.i.	n.i.	n.i.	n.i.	n.i.	n.i.	n.i.	n.i.	n.i.	n.i.	n.i.	n.i.	n.i.	n.i.	n.i.	n.i.	n.i.	n.i.	n.i.	3.17	n.i.	n.i.	1.1
Amphenicols	Chloramphenicol	n.i.	n.d.	0.03	n.d.	n.d.	0.06	n.i.	0.6	0.43	0.15	n.d.	n.d.	22.83	n.d.	n.i.	n.d.	n.d.	n.d.	n.d.	n.d.	n.i.	n.i.	n.d.	n.i.	n.d.
	Thiamphenicol	n.i.	n.d.	n.d.	n.d.	n.d.	0.07	n.i.	0.02	0.25	0.25	n.d.	n.d.	n.i.	n.i.	n.i.	n.d.	n.d.	n.d.	n.i.	n.i.	n.i.	n.i.	n.d.	n.i.	n.i.
	Florfenicol	n.i.	0.19	1.2	1.57	0.32	0.05	n.i.	0.4	0.31	0.03	n.d.	1.31	n.d.	5.06	n.i.	9.9	12.1	13.0	0.3	n.i.	n.i.	n.i.	4.36	n.i.	n.d.
	Florfenicol amine	n.i.	n.i.	n.i.	n.i.	n.i.	n.i.	n.i.	n.i.	n.i.	n.i.	n.i.	n.i.	n.i.	n.i.	n.i.	n.i.	n.i.	n.i.	n.d.	n.i.	n.i.	n.i.	n.i.	n.i.	n.i.
Miscellaneous	Lincomycin	n.i.	n.i.	n.i.	n.d.	n.i.	n.i.	n.d.	n.i.	n.i.	n.i.	n.i.	n.d.	1.5	n.d.	n.i.	n.d.	n.d.	n.d.	n.d.	n.i.	n.i.	n.i.	n.d.	n.i.	n.d.
	Cyadox	n.i.	n.i.	n.i.	n.d.	n.i.	n.i.	n.i.	n.i.	n.i.	n.i.	n.i.	n.d.	n.i.	n.d.	n.i.	n.d.	n.d.	n.d.	n.d.	n.i.	n.i.	n.i.	n.d.	n.i.	n.i.
	Quinocetone	n.i.	n.i.	n.i.	n.d.	n.i.	n.i.	n.i.	n.i.	n.i.	n.i.	n.i.	n.d.	n.i.	n.i.	n.i.	n.i.	n.i.	n.i.	n.i.	n.i.	n.d.	n.i.	n.i.	n.i.	n.i.
	Olaquindox	n.i.	n.i.	n.i.	n.i.	n.i.	n.i.	n.i.	n.i.	n.i.	n.i.	n.i.	n.i.	n.i.	n.i.	n.i.	n.i.	n.i.	n.i.	n.i.	n.i.	n.i.	n.i.	n.i.	n.i.	n.i.
	Metronidazole	n.i.	n.i.	n.i.	n.i.	n.i.	n.i.	n.i.	n.i.	n.i.	n.i.	n.i.	n.i.	n.i.	n.i.	n.i.	n.i.	n.i.	n.i.	n.i.	n.i.	n.i.	n.i.	n.i.	n.d.	n.i.

*Note*: Data compiled from real‐life biomonitoring studies from urine, feces, and serum. Article information: (1) Zhou, Cuasquer, et al. (2021), (2) Wang et al. (2018) (Adult)*, (3) Wang et al. (2018) (Children)*, (4) Geng et al. (2020)*, (5) Wang et al. (2021)*, (6) Zeng et al. (2020)*, (7) Li et al. (2017), (8) Wang, Wang, Wang, Fang, et al. (2016)*, (9) Zhou, Zhu, et al. (2021) (Children)*, (10) Zhou, Zhu, et al. (2021) (Pregnant women)*a, (11) Zhang et al. (2022), (12) Geng et al. (2021)*, (13) Liu et al. (2021)*, (14) Liu et al. (2021)*, (15) Wang et al. (2020), (16) Zhang et al. (2020) (Children), (17) Zhang et al. (2020) (Adult), (18) Zhang et al. (2020) (Elderly adult), (19) Liu et al. (2020)*, (20) Huang et al. (2020)*, (21) Huang et al. (2019), (22) Wang et al. (2019), (23) Zhu et al. (2020), (24) Bekoe et al. (2020), and (25) Liu et al. (2017). An asterisk indicates that the 95^th^ percentile was reported where available.

Abbreviations: A, adult; C, children; EA, elderly adult; GP, general population; n.d., not detected; n.i., not investigated; PW, pregnant women.

^a^ First trimesters of pregnancy.

**TABLE 3:** Article information and detection frequencies (%) of different veterinary drugs/antibiotic groups in human biofluids compiled from real‐life biomonitoring studies.

Study information					Detection frequencies % (veterinary drugs/antibiotic groups)					
Author information	Population type	Biofluid samples	LC method	Sample size (*n*)	Sulfonamides	Macrolides	β‐Lactams	Tetracyclines	Fluoroquinolones	Amphenicols
Zhou, Cuasquer, et al. (2021)	Adult	Urine	LC–MS/MS	30	n.p.	n.p.	n.p.	n.p.	n.p.	n.p.
Wang et al. (2018)^a^	Adult	Urine	LC–Q‐ToF	822	18.1	2.1	n.i.	3.7	26.8	10.9
Wang et al. (2018)^a^	Children	Urine	LC–Q‐ToF	284	17.6	7.7	n.i.	10.2	19.4	31.7
Geng et al. (2020)^a^	Pregnant women	Urine	LC–MS/MS	9136	64.0	13.0	24.6	47.8	65.6	31.9
Wang et al. (2021)^a^	Children	Urine	LC–Q‐ToF	1134	13.5	5.8	n.i.	9.5	27.2	19.0
Zeng et al. (2020)^a^	Pregnant women	Urine	LC–MS/MS	762	78.5	8.5	n.i.	n.p.	94.0	36.5
Li et al. (2017)	Children	Urine	LC–MS/MS	31	n.p.	n.p.	n.p.	n.p.	n.p.	n.i.
Wang et al. (2016)^a^	Children	Urine	LC–Q‐ToF	393	34.4	19.6	7.1	6.9	28.5	46.6
Zhou, Zhu, et al. (2021)^a^	Children	Urine	LC–MS/MS	107	n.i.	n.i.	n.i.	35.5	n.i.	20.6
Zhou, Zhu, et al. (2021)^a^	Pregnant women	Urine	LC–MS/MS	126	n.i.	n.i.	n.i.	27.0	n.i.	5.6
Zhang et al. (2022)	Pregnant women	Urine	LC–MS/MS	735	78.9	7.9	n.i.	27.3	93.9	n.i.
Geng et al. (2021)	Pregnant women	Urine	LC–MS/MS	2287	n.p.	n.i.	n.i.	n.p.	n.p.	n.p.
Liu et al. (2021)^a^	General population	Urine	LC–MS/MS	87	46.0	20.7	n.i.	46.0	26.4	13.8
Liu et al. (2021)^a^	Elderly adult	Urine	LC–MS/MS	713	55.7	29.5	26.4	43.1	48.2	23.6
Wang et al. (2020)	General population	Feces	LC–MS/MS	180	n.p.	n.p.	n.p.	n.p.	n.p.	n.p.
Zhang et al. (2020)	Children	Urine	LC–MS/MS	326	49.7	37.1	31	33.7	49.6	29.5
Zhang et al. (2020)	Adult	Urine	LC–MS/MS	263	45.6	36.5	24	47.5	53.2	24.3
Zhang et al. (2020)	Elderly adult	Urine	LC–MS/MS	102	39.2	28.4	19.6	38.2	49.0	20.6
Liu et al. (2020)^a^	Pregnant women	Urine	LC–MS/MS	302	33.1	11.6	10.9	40.4	52.6	16.9
Huang et al. (2020)^a^	Children	Urine	LC–MS/MS	249	3.6	16.1	8.03	24.1	32.5	1.6
Huang et al. (2019)	Children	Urine	LC–MS/MS	6	n.p.	n.p.	n.i	n.p.	n.p.	n.i.
Wang et al. (2019)	Adult	Urine	LC–MS/MS	12	n.i.	n.i.	n.i.	n.i.	n.p.	n.i.
Zhu et al. (2020)^a^	Elderly adult	Urine	LC–MS/MS	990	55.8	28.5	25.6	44.4	50.0	24.9
Bekoe et al. (2020)	Adult	Urine	LC–MS/MS	177	n.p.	n.p.	n.p.	n.p.	n.p.	n.i.
Liu et al. (2017)	Adult	Serum	LC–MS/MS	107	30.8	6.5	7.5	2.8	25.2	2.8

*Note*: Data compiled from real‐life biomonitoring studies in urine, feces, and serum.

Abbreviations: n.i., not investigated; n.p., not provided.

^a^ The 95% CI metric was used for further calculations of detection frequencies (DF%).

In the reviewed literature, detection frequencies and concentration ranges were highly heterogeneous for individual antibiotics as well as for whole classes of antibiotics. For instance, azithromycin and erythromycin were the most frequently detected macrolide antibiotics with urine concentrations in the range of 0.03–62 and 0.2–78 ng/mL, whereas urine levels in the range of 0.37–40, 1.54–12.5, 0.14–105, and 0.19–1.26 ng/mL were reported for the tetracyclines oxytetracycline, doxycycline, tetracycline, and chlortetracycline, respectively (Table 2). Ciprofloxacin, ofloxacin, enrofloxacin, and norfloxacin were the most frequently detected fluoroquinolones, and urine levels were in the concentration ranges of 0.058–23.8, 0.29–54.7, 0.082–2.8, and 0.14–4.5 ng/mL, respectively. Moreover, trimethoprim (0.25–2.0 ng/mL), sulfamethoxazole (0.11–2.82 ng/mL), sulfaclozine (17.9–23.7 ng/mL), sulfadiazine (0.038–0.26 ng/mL), and sulfamethazine (0.17–5.25 ng/mL) were under the most frequently detected sulfonamide antibiotics in human biofluids. Finally, amoxicillin and penicillin V are common β‐lactam antibiotics with reported concentrations in the range of 0.98–15.7 and 1.9–5.73 ng/mL, respectively, whereas florfenicol (0.03–5.0 ng/mL) was the most frequently detected amphenicol antibiotic, followed by chloramphenicol (0.03–22.83 ng/mL). In general, it can be stated that antibiotic residues are detectable in considerable fractions of investigated population groups. For instance, Wang et al. (2020) showed that the overall detection frequency of antibiotic compounds is approximately 50% in feces samples from a Chinese population of children, adults, and elderly people, with sulfadimidine (90%) being the most frequently detected antibiotic compound, whereas ampicillin showed the highest reported maximum concentration (49.5 µg/kg).

Higher concentrations of typical veterinary drugs/antibiotics were observed in adults, whereas human antibiotics were higher in children (Zhang et al., 2020). Delineating the origin of various compounds is challenging, due to potential applications in both veterinary and human medicine. Medical prescription of antibiotics for children is frequent as they are more prone to various diseases due to their not fully matured immune systems and organs compared to adults (Zhang et al., 2020). In a recent study, Wang et al. (2020) analyzed stool samples and presented similar findings indicating that intentional medical use of antibiotics is a primary exposure pathway for children, whereas environmental sources are more relevant for adults. Detection frequencies of VAs in feces were higher in adults (20%) compared to antibiotics for human use only (1.7%), and an association of the economic status with higher antibiotic levels (i.e., higher exposure levels in economically less developed areas) was reported (Wang et al., 2020). Antibiotic groups such as sulfonamides, tetracyclines, and quinolones are extensively used for animal treatments and can enter into the human food chain through water, soil, and food of animal origins. Higher detection frequencies and concentrations of VAs in feces may suggest that environmental exposure through water and food is the most likely cause of antibiotic ingestion route in the adult gut. Despite being underrepresented in HBM studies, VAs may significantly impact human health, and further research is needed to shed light on the detailed mechanism by which residual antibiotics affect human health including their influence on the gut microbiome. To conclude, it is essential to precisely assess the co‐exposure effects of multiple antibiotics together with other environmental chemicals during exposome studies for linking exposure to health conditions (e.g., allergy or obesity) or other disease outcomes (e.g., cancer).

## 6 CO‐EXPOSURE TO MULTIPLE ANTIBIOTICS AND ASSOCIATED PUBLIC HEALTH RISKS

Several research studies investigated potential routes of (co‐)exposure in various populations and observed associations between exposure to multiple classes of antibiotics and potential public health risks such as increased tendencies to develop obesity, eczema, depression, or other mental disorders (Geng et al., 2021; Liu et al., 2021; Wang, Wang, Wang, Fang, et al., 2016; Zhang et al., 2021). A health risk assessment study was performed to determine 18 antibiotics and their association with demographic profiles, that is, age, gender, geography, body mass index (BMI), and dietary patterns (Liu et al., 2017). Diet, in particular, animal‐based products such as meat, milk, and eggs, has a significant association with the presence of antibiotics in urine. A total of 17 antibiotics were found in urine, with doxycycline being the predominantly detected antibiotic and 9% of the population having a hazard index (HI) >1. In contrast to the relationship between animal product‐rich diets and antibiotic levels, no association between antibiotic levels and vegetable‐ or fresh‐fruit‐based diets was observed (Liu et al., 2022). Wang, Yang, et al. (2018) showed that sex, age, and family income were correlated with the detection frequency of antibiotics. Moreover, children who consumed aquatic products, meat, milk, or dairy products frequently showed higher detection frequencies of antibiotics. In total, 6% of the children had an HI >1, and the fluoroquinolone antibiotics ciprofloxacin and ofloxacin had a hazard quotient (HQ) >1. These findings revealed that a potential health risk persists for children due to exposure to antibiotics. The same group reported a similar observation for children and pregnant women, with 5.6% of adults showing HI/HQ >1 and ciprofloxacin being the major contributing antibiotic (Wang, Yang, et al., 2018). To understand the effect of environmental antibiotic exposure on fetal and infant growth, a pre‐ and postnatal biomonitoring study has been conducted on 735 pregnant women and their offspring (Zhang et al., 2022). In this study, children's growth parameters, that is, height and weight, were measured at birth and 12, 24, and 60 months after birth. The authors could demonstrate that maternal urinary antibiotic concentration was inversely associated with birth weight and ponderal index, with ciprofloxacin and florfenicol being the main contributors to reduced fetal growth. In a similar study, maternal antibiotic concentrations were investigated to evaluate health risks in pregnant women in a Chinese population (Zeng et al., 2020). Results indicated that sulfonamides and fluoroquinolones were the most abundant antibiotic classes in urine, with ciprofloxacin as the predominant antibiotic compound, followed by norfloxacin. In this study, high VA exposure was associated with frequent consumption of animal products (milk and egg) during pregnancy. Interestingly, a high intake of seafood and meat (livestock and poultry) showed no significant association with higher antibiotic exposures in this study (Zeng et al., 2020).

In another study, Zhu et al. (2020) monitored 45 antibiotics in samples from an elderly population, detecting at least one of 34 investigated antibiotics in 93% of all urine samples (detection frequencies of each antibiotic varied from 0.2% to 35%) and showing that 6.7% of the investigated individuals may suffer from health risks related to alterations of gut microbiota caused by VAs, especially by exposure to ciprofloxacin. These findings are also supported by a study by Zhang et al. (2020) that showed the detection of antibiotics in 92% of urine samples from three generations of a Chinese population. In this study, 45 antibiotics (parent compounds and two metabolites) were investigated, and 34 were detected in urine. Interestingly, the exposure profile differed between the three investigated generations: parents showed higher detection frequencies for penicillin V, chlortetracycline, doxycycline, enrofloxacin, and ciprofloxacin, whereas tetracycline, danofloxacin, and ofloxacin were mostly found in grandparents. Ciprofloxacin was the predominant antibiotic in samples from all three generations. An HI >1 was observed in 14% of children, 24% of parents, and 12% of grandparents (Zhang et al., 2020).

Early life exposure is of central research interest, as several studies showed associations between antibiotic exposure and an increased risk of obesity in children (Wang et al., 2016). Various antibiotics that are suspected to originate from food and water were recently investigated in urine samples of >500 children. Thus, 21 antibiotics from six antibiotic classes were detected between 35% and 80% of the participants, and a significant association was observed between exposure to antibiotics and obesity (Wang, Wang, Wang, Fang, et al., 2016). In addition, other health impacts could be associated with early‐life exposure to antibiotics. In a prospective birth cohort study, associations between prenatal low‐dose antibiotic exposures at different time points and allergic disease in children at the age of 4 years were investigated. Detection frequencies of nine antibiotics were >10% regardless of the trimester, and at least one antibiotic was detected in >90% of all samples regardless of the investigated trimester. Moreover, an association between maternal antibiotic exposure and the risk for the development of eczema and asthma was observed. Especially, exposure to sulfamethazine in the first trimester, ciprofloxacin in the second trimester, and oxytetracycline in the third trimester of pregnancy was reported to be relevant for disease development later (Geng et al., 2021). In addition to impairing physical health, environmental and/or dietary exposure to antibiotics may also affect children's mental health. Zhang et al. (2021) examined the association between urinary antibiotic exposures and mental disorders in a community‐based cross‐sectional study of 278 children, and ciprofloxacin could be associated with an increased risk of mental disorders developing for both lower (odd ratio [OR] = 4.06, 95% CI: 1.69–9.78) and higher (OR = 6.04, 95% CI: 2.59–14.08) antibiotic concentrations.

Furthermore, Liu et al. (2021) showed that high antibiotic levels are associated with an increased risk for the development of depression. In this study, 45 antibiotics were measured in urine samples from elderly people in China. In total, 34 antibiotics were observed in 93% of the samples, and some antibiotics even exceeded concentrations of 10,000 ng/mL. Multinominal logistic regression showed an increased risk of depression associated with concentrations of azithromycin (OR = 1.81, 95% CI: 1.09–3.0) and sulfaclozine (OR = 1.54, 95% CI: 1.02–2.28). Notably, sex‐ and age‐specific differences were also observed between antibiotic exposure and depression. In males, higher VA concentrations (OR = 2.04, 95% CI: 1.13–3.71) were associated with an increased risk of depression, with sulfaclozine showing a particularly strong positive correlation (OR = 2.10, 95% CI: 1.15–3.82). Females showed an association between depression and all investigated antibiotics, particularly azithromycin (OR = 2.25, 95% CI: 1.20–4.21), norfloxacin (OR = 2.41, 95% CI: 1.01–5.78), and tetracyclines (OR = 1.74, 95% CI: 1.04–2.85). Participants under the age of 70 had a significantly stronger association (OR = 2.64, 95% CI: 1.03–6.79) between antibiotic exposure and depression than those over the age of 70. Another study also demonstrated that recurrent antibiotic exposure was associated with an increased risk of depression (Lurie et al., 2015). Concluding, this evidence highlights the importance of improving our knowledge about the co‐exposure scenarios of VAs in urine, plasma, and stool samples.

## 7 CO‐EXPOSURE OF ANTIBIOTICS AND OTHER ENVIRONMENTAL CONTAMINANTS

Humans are exposed to a cocktail of environmental exposures daily due to individual dietary habits, lifestyle, and the (social) environment (Ayeni et al., 2022; Cortejade et al., 2016). Chronic exposure to low doses of VAs can reach the human body through the ingestion of foods of animal origin (e.g., meat, milk, and egg) if the withdrawal period (minimum time between the last administration of antibiotics and the production of meat or other animal‐derived food products) is not widely monitored and regulated. Strict adherence to withdrawal periods can ensure that food is safe for consumption and that VAs do not exceed maximum residue limits (MRLs). Chronic exposure to VA residues at low doses may cause health risks in humans. Therefore, the combined effects of VAs and various other environmental chemicals should be thoroughly investigated on the exposome scale to fully understand the harmful effects on human health mediated by cocktails of VAs and other relevant environmental contaminants. Conceptionally, exposomics aims to address all environmental exposures an individual experiences over the entire lifespan (Kalia et al., 2022). As such, the integration of new technologies with other established omics approaches may allow to investigate exogenous compounds along with endogenous metabolites reflecting the biological response to environmental perturbations (Flasch, Fitz, et al., 2022; Lai et al., 2024; Verri Hernandes & Warth, 2024). Therefore, ongoing improvements, adaptions, and extensions of the toolbox of established methods are necessary to match the complexity of the addressed problem. Given the highlighted relevance, the full integration of VAs into HBM studies appears to be a promising approach to shed light on the combined effects of VA exposure with other relevant environmental chemicals. This further would allow us to characterize risks and implement regulations and preventive measures to improve human health. Although VAs appear to be a relevant compound class for HBM studies, a routine integration into the exposome framework is not yet achieved, and the number of HBM studies integrating VA analysis with other environmentally relevant compounds is still limited. In general, analytical workflows used to analyze environmental chemicals in human biofluids are comparable to the ones used for VA analysis and typically include analyte extraction/protein precipitation, sample cleanup steps, chromatographic separation, and detection using MS analyzers (Yao et al., 2018). A recent example of our group demonstrated the technical feasibility of scaling up broad‐scope, targeted HBM methods by integrating vetarinary drugs (Hossain et al., 2024).

Besides targeted assays, HRMS‐based targeted, suspect, and nontargeted screening approaches are increasingly popular in exposome‐type HBM studies. For example, a total of 38 environmental chemicals including pesticides, VAs, and parabens were identified and quantified at the nanograms per milliliter level in human urine using LC–QToF (Cortejade et al., 2016). In another recent co‐exposure study, Wang et al. (2021) determined the presence of 23 urinary antibiotics along with bisphenol A (BPA) and monobutyl phthalate (MBP) in urine samples from school children and assessed temporal trends of antibiotic exposure between the years of 2017 and 2020. Interestingly, antibiotics were detected in 52% of all urines, and levels declined between the years 2017 and 2020, whereas no correlation was observed with BPA and MBP levels or with demographic factors, food, or water consumption (Wang et al., 2021). In a recent HRMS study, several antibiotics including amoxicillin, oxytetracycline, and azithromycin have been detected in infant's feces, and their presence can be linked to therapeutic use or breastfeeding (Oesterle et al., 2024).

## 8 INTEGRATION OF VAs INTO THE EXPOSOME FRAMEWORK: CURRENT SCENARIO AND FUTURE PERSPECTIVES

VAs are predominantly consumed in the livestock industry, and 70%–80% of the global antibiotics are used for animal production (World Economic Forum, 2023) and the EU has established MRLs for major antibiotic groups in animal food products (EU, 2010). The presence of VAs in foods of animal origin results in potential risks to consumers, as indicated by the summarized literature, and residual antibiotics are potentially linked with a wide range of adverse effects on human health, including obesity, allergy, mental disorders, and depression. Thus, the summarized evidence highlights the importance of full implementation of large‐scale exposure and risk assessment study in humans (Geng et al., 2021; Liu et al., 2017, 2021; Wang, Wang, Wang, Fang, et al., 2016; Zhang et al., 2021). Additionally, most of the studied antibiotics in published HBM studies (see Table 2) were frequently detected in vulnerable (sub)populations, including children, elderly people, or pregnant women. However, applicable guidelines and regulations for HBM of residual (veterinary) antibiotics in human biofluids in the EU are largely missing. Despite the considerable number of studies highlighting the presence of residual antibiotics in human biofluids, most of which are based on data from Chinese sample cohorts, VAs are not yet incorporated in main EU‐wide HBM studies, risk and exposure assessments, or relevant exposome platform projects. These projects (HELIX, HEALS, EXPOsOMICS, and HBM4EU) covered a total of 59, 98, seven, and 230 environmental chemicals, respectively (Huhn et al., 2021) and focused on other priority‐based listed substances. For instance, bisphenols, mycotoxins, pesticides, lead, mercury, cadmium, arsenic, chromium, acrylamide, phthalates, polycyclic aromatic hydrocarbons, flame retardants, or benzophenones were analyzed as part of HBM4EU, whereas VAs were not included (Huhn et al., 2021). A possible explanation is the traditional discipline barriers, that is, the frequent separation between food‐related and environmental research focus. Considering the ubiquitous presence of residual antibiotics in various HBM studies and related adverse effects on human health, a full incorporation of VAs into the current large‐scale human exposome framework seems to be justified. This would allow us to fill research gaps and to further evaluate the combined effects of various VAs and other environmental chemical exposures for a better understanding of the chemical exposome and its health effects, for example, by identifying novel biomarkers of exposure.

Measuring the chemical exposome is a complex task that requires broad and sensitive analytical methods to cover VAs combined with other contaminants or environmental chemicals. So far, only a few HBM studies have investigated VAs together with other environmental chemicals, including pesticides, parabens, bisphenol, or phthalates (Cortejade et al., 2016; Wang et al., 2021; Yao et al., 2018). Therefore, the development of targeted and nontargeted LC–MS methods in HBM programs to characterize large panels of antibiotics together with other major environmental chemicals will greatly help to understand the potential associations between combined environmental exposures and human health risks. Exposome‐scale investigations using nontargeted analysis can be expanded to assess various environmental exposures (e.g., metabolites, toxicants, xenobiotics, biotransformation products, etc.) and their combinatory effects on human health (Warth et al., 2017). Therefore, holistic and systematic LC–MS‐based exposomics/metabolomics workflows, including advanced bioinformatics tools, will be needed to further extend the total analyte coverage toward additional VAs and environmental chemicals. However, despite recent instrumental advances of LC–HRMS‐based, targeted (multianalyte) assays, LC–MS/MS still shows superior instrument sensitivity and allows to report fully quantitative data at trace levels. Thus, such methods should be regarded as the current gold standard for HBM studies (Flasch et al., 2023). Expanding the coverage of currently employed analytical methods for novel markers of exposure is an ongoing process. For instance, an exposome‐scale targeted LC–MS/MS method validated for the simultaneous determination of >80 highly diverse environmental chemicals, including plasticizers, phytoestrogens, mycoestrogens, personal care product ingredients, industrial chemicals, food‐processing products, or phytotoxins (Jamnik et al., 2022), was recently expanded to additionally include several VAs and pesticides (Hossain et al., 2024). Moreover, comprehensive untargeted LC–HRMS‐based workflows for combined exposomics and metabolomics analysis are expected to play a more prominent role in future exposome research. Such assays provide powerful tools to allow us to assess chemical exposure and the (metabolic) biological response in parallel using nontargeted analysis and suspect screening (Flasch, Fitz, et al., 2022). Such generic workflows would allow the retrospective analysis of environmental chemicals and their metabolites in human samples. Moreover, they may enable us to identify exposure risk factors and associated biomarkers of biological perturbations, including potential toxicological effects (Flasch, Fitz, et al., 2022; Verri Hernandes & Warth, 2024). A full integration of multiomics workflows may provide a deep and systemic understanding of toxicity, adverse outcome pathways, or the identification of endogenous chemical signatures serving as early biomarkers for health effects and biological perturbations caused by exposure to VAs and other relevant chemicals. To incorporate the exposome component in such multiomics studies, a comprehensive and systematic approach utilizing untargeted HRMS is necessary in order to deliver high‐dimensional environmental data (as the “exposome layer”) into a multiomics framework (Miller, 2021).

Finally, a detailed assessment of the combined effects of various parent VAs and their biotransformation products would be a prerequisite to fully address and characterize the health effects of VA exposures. For instance, recently described isotope‐assisted untargeted workflow to elucidate the fate of xenoestrogens and food toxins could be adopted to identify so‐far unknown antibiotic metabolites and biotransformation products in human cell models (Flasch et al., 2020; Flasch, Bueschl, et al., 2022). In this sense, novel state‐of‐the‐art LC–MS/MS and LC–HRMS workflows and suitable bioinformatic tools are necessary to shed light on multiclass co‐exposures including VAs and their biotransformation products.

## 9 CONCLUSIONS

Veterinary drugs and antibiotics are an integral part of our overall xenobiotic burden. However, they are infrequently assessed by current HBM and exposomics research and the corresponding analytical workflows. In this review, we outlined that various VAs have been detected at significant levels in human biofluids and that detection frequencies of specific groups of antibiotics reached >90% in certain studies. It is noteworthy that the majority of the studied populations in the reviewed HBM studies were conducted in China. Despite China being the world's largest producer and user of antibiotics, the EU consumed a large volume of veterinary medicinal products for food‐producing animals. Therefore, more HBM studies are needed for the parallel monitoring of multiple classes of antibiotics and their metabolites and to identify potential risks to human health within the EU and other countries. Presently, the Center for Disease Control and Prevention conducted the most comprehensive biomonitoring program, which covers 319 substances in blood and urine, whereas HBM4EU has prioritized 230 individual chemicals and has a plan to expand the coverage of suspect or emerging chemicals to develop targeted HBM methods for the prioritized chemical substances (Huhn et al., 2021). Although there is still a long way to go to cover all exposure chemicals and their metabolites, VAs and their biotransformation products can be regarded as a relevant analyte class of the chemical exposome, and their inclusion in large‐scale HBM and exposure assessment studies should be considered to obtain a more comprehensive view of the chemical exposome and its mixture effects on human health.

Full integration of novel analytical methods and workflows into population‐scale exposure studies based on VAs is a prerequisite to obtaining a comprehensive picture of antibiotic exposures and health effects. Furthermore, a systematic exposome workflow to detect VAs alongside other environmental chemicals and evaluate potential health risks is lacking to date. Most existing targeted MS/MS workflows typically detect a few dozen to less than 100 environmental chemicals per analytical run. However, most antibiotics that are associated with human health risks are typically not included in such LC–MS/MS workflows. Therefore, targeted LC–MS/MS exposome approaches need to be further expanded. Finally, the implementation of LC–HRMS workflows for nontargeted analysis and suspect screening, including suitable bioinformatics pipelines, promises to further explore additional emerging chemicals as a part of the exposome.

To conclude, the exposome is an emerging concept that has significantly evolved during the last decade. Recent evidence of VA exposure and its related health risks justifies further integration of those compounds into exposomics assays. The assessment of mixture effects with diverse environmental chemicals is needed to further improve our understanding of their contribution to disease etiology. Advanced LC–MS/MS and LC–HRMS technologies offer a vast potential to fill existing research gaps in the area of antibiotic exposure, metabolism, and health effects.

## AUTHOR CONTRIBUTIONS

**Md Zakir Hossain**: Conceptualization; funding acquisition; writing—original draft; visualization; project administration; data curation. **Max L. Feuerstein**: Writing—review and editing; data curation. **Benedikt Warth**: Conceptualization; funding acquisition; project administration; data curation; supervision; writing—original draft.

## CONFLICT OF INTEREST STATEMENT

The authors declare no conflicts of interest.
